# Contributions of event rates, pre-hospital deaths, and deaths following hospitalisation to variations in myocardial infarction mortality in 326 districts in England: a spatial analysis of linked hospitalisation and mortality data

**DOI:** 10.1016/S2468-2667(22)00108-6

**Published:** 2022-07-16

**Authors:** Perviz Asaria, James E Bennett, Paul Elliott, Theo Rashid, Hima lyathooray Daby, Margaret Douglass, Darrel P Francis, Daniela Fecht, Majid Ezzati

**Affiliations:** Department of Epidemiology and Biostatistics, MRC Centre for Environment and Health, School of Public Health, Imperial College London, London, UK; Department of Cardiology, Imperial College NHS Trust, London, UK; Department of Epidemiology and Biostatistics, MRC Centre for Environment and Health; Department of Epidemiology and Biostatistics, MRC Centre for Environment and Health, UK Small Area Health Statistics Unit; Department of Epidemiology and Biostatistics, MRC Centre for Environment and Health; Department of Epidemiology and Biostatistics, MRC Centre for Environment and Health, UK Small Area Health Statistics Unit; Department of Epidemiology and Biostatistics, MRC Centre for Environment and Health, UK Small Area Health Statistics Unit; School of Public Health, Imperial College London, London, UK; Department of Cardiology, Imperial College NHS Trust, London, UK, National Heart and Lung Institute, Imperial College London, London, UK; Department of Epidemiology and Biostatistics, MRC Centre for Environment and Health, UK Small Area Health Statistics Unit; Department of Epidemiology and Biostatistics, MRC Centre for Environment and Health, Regional Institute for Population Studies, University of Ghana, Accra, Ghana

## Abstract

**Background:**

Myocardial infarction mortality varies substantially within high-income countries. There is limited guidance on what interventions—including primary and secondary prevention, or improvement of care pathways and quality—can reduce myocardial infarction mortality. Our aim was to understand the contributions of incidence (event rate), pre-hospital deaths, and hospital case fatality to the variations in myocardial infarction mortality within England.

**Methods:**

We used linked data from national databases on hospitalisations and deaths with acute myocardial infarction (ICD-10 codes I21 and I22) as a primary hospital diagnosis or underlying cause of death, from Jan 1, 2015, to Dec 31, 2018. We used geographical identifiers to estimate myocardial infarction event rate (number of events per 100 000 population), death rate (number of deaths per 100 000 population), total case fatality (proportion of events that resulted in death), pre-hospital fatality (proportion of events that resulted in pre-hospital death), and hospital case fatality (proportion of admissions due to myocardial infarction that resulted in death within 28 days of admission) for men and women aged 45 years and older across 326 districts in England. Data were analysed in a Bayesian spatial model that accounted for similarities and differences in spatial patterns of fatal and non-fatal myocardial infarction. Age-standardised rates were calculated by weighting age-specific rates by the corresponding national share of the appropriate denominator for each measure.

**Findings:**

From 2015 to 2018, national age-standardised death rates were 63 per 100 000 population in women and 126 per 100 000 in men, and event rates were 233 per 100 000 in women and 512 per 100 000 in men. After age-standardisation, 15·0% of events in women and 16·9% in men resulted in death before hospitalisation, and hospital case fatality was 10·8% in women and 10·6% in men. Across districts, the 99th-to-1st percentile ratio of age-standardised myocardial infarction death rates was 2·63 (95% credible interval 2·45–2·83) in women and 2·56 (2·37–2·76) in men, with death rates highest in parts of northern England. The main contributor to this variation was myocardial infarction event rate, with a 99th-to-1st percentile ratio of 2·55 (2·39–2·72) in women and 2·17 (2·08–2·27) in men across districts. Pre-hospital fatality was greater than hospital case fatality in every district. Pre-hospital fatality had a 99th-to-1st percentile ratio of 1·60 (1·50–1·70) in women and 1·75 (1·66–1·86) in men across districts, and made a greater contribution to variation in total case fatality than did hospital case fatality (99th-to-1st percentile ratio 1·39 [1·29–1·49] and 1·49 [1·39–1·60]). The contribution of case fatality to variation in deaths across districts was largest in women aged 55–64 and 65–74 years and in men aged 55–64, 65–74, and 75–84 years. Pre-hospital fatality was slightly higher in men than in women in most districts and age groups, whereas hospital case fatality was higher in women in virtually all districts at ages up to and including 65–74 years.

**Interpretation:**

Most of the variation in myocardial infarction mortality in England is due to variation in myocardial infarction event rate, with a smaller role for case fatality. Most variation in case fatality occurs before rather than after hospital admission. Reducing subnational variations in myocardial infarction mortality requires interventions that reduce event rate and pre-hospital deaths.

**Funding:**

Wellcome Trust, British Heart Foundation, Medical Research Council (UK Research and Innovation), and National Institute for Health Research (UK).

## Introduction

Mortality from ischaemic heart disease has declined substantially in high-income countries, driven by both a decline in incidence and improved survival of myocardial infarction—the acute presentation of ischaemic heart disease which has the potential to be rapidly fatal in the absence of appropriate interventions.^1,2^ This decrease in incidence of myocardial infarction has been due to reductions in risk factors such as smoking, blood pressure, and cholesterol in the population, as well as primary and secondary prevention through pharmacological treatment in individuals at high risk.^3^ Improvement in myocardial infarction survival has been achieved by more rapid diagnosis and revascularisation and through the use of anti-platelet agents based on evidence from randomised trials. At the health-system level, the establishment of cardiology wards, coronary care units, and cardiac intensive care units, staffed by specialist cardiac doctors and nurses, has helped to standardise and optimise the delivery of the aforementioned treatments and to identify and intervene on complications early.^4^

Despite these aggregate successes, ischaemic heart disease mortality varies markedly within high-income countries, including in England, and the available data indicate that mortality due to myocardial infarction is a major contributor to this variation.^5–7^ Myocardial infarction mortality, and its variation within a population, can be reduced through primary and secondary prevention measures to reduce event rates; improving awareness of myocardial infarction symptoms and initial response time to reduce the share of patients with myocardial infarction who die before reaching a hospital; and improving hospital care. Many current trial and standardisation efforts are targeted towards the latter component.^4^ However, there are limited data on the relative importance of these three contributors to subnational variations in myocardial infarction mortality, which are needed to inform the selection of optimal strategies for reducing myocardial infarction mortality where it is high.

We used linked data on hospitalisations and deaths in England’s 326 local authority districts (political and administrative units that are used for the allocation of public health and social care budgets and for the formulation and delivery of primary prevention; referredto henceforth as districts) to determine how much the geographical variation in myocardial infarction mortality arises from variations in event rates and in case fatality and its constituents, namely pre-hospital death and death following hospitalisation (referred to as hospital case fatality).

## Methods

### Data sources

We used data on hospitalisations due to myocardial infarction from the Hospital Episode Statistics (HES) database (which contains information on all admissions to the National Health Service, a publicly funded health-care system that serves all of England’s residents), and data on deaths due to myocardial infarction from the Office for National Statistics (ONS) database (which records all deaths in England) for the years 2015–18. These two data sources provide information on the numbers of deaths and admissions for myocardial infarction and other diseases in the prespecified period. Their linkage allows the identification of myocardial infarction admissions that resulted in death, and allows the separation of myocardial infarction deaths that followed a (recent) admission for any cause from those that occurred with no (recent) admission to hospital. Both sources include information on age, sex, and postcode of residence, which was used to assign events and deaths to local authority districts. Population data by age, sex, district, and year were from the ONS.^8^ The median population of a district in 2018 was 133 473 (IQR 99 555–210 035), with a median 62 936 residents (48022–88474) per district aged 45 years or older (the focus of this analysis).

The HES and the HES–ONS linked mortality data were provided by NHS Digital. Linkage was extended to 28 days before Jan 1, 2015, and 28 days after Dec 31, 2018, so that linked events at the beginning and end of the analysis period were captured. The process of linkage is imperfect; in England, approximately 97·6% of deaths recorded in hospitalisation data match civil registration records, and 2·4% do not match.^9^ Failure to match can be due to deaths referred to coroners for inquest or to unsuccessful linking (itself due to missing or inaccurately recorded patient identifiers).

HES and ONS use ICD-10 codes for recording diagnoses and cause of death. Our primary outcome was acute myocardial infarction (ICD-10 codes I21 and I22) as a primary hospital diagnosis or cause of death. These codes capture acute myocardial infarction, regardless of whether it is a first or subsequent event. We excluded ICD-10 codes I20 and I23–I25, which comprise angina, complications of myocardial infarction, and chronic or old atherosclerotic disease, and which do not necessitate the same interventions as acute myocardial infarction.

HES data are recorded by finished consultant episodes (FCEs), such that a transfer of patient care between physicians results in a new FCE. We collapsed adjacent FCEs into continuous spells of care using a standard grouping algorithm.^10^ Acute myocardial infarction has a duration of 28 days or less from onset within the ICD-10 system.^11^ We treated transfers between hospitals occurring within 2 days and readmissions within 28 days of each other as part of the same spell of care, so that each myocardial infarction event was counted only once. A myocardial infarction occurring more than 28 days after a previous myocardial infarction was counted as a new event. We use the terms event or admission hereafter to refer to a continuous spell of care for a single myocardial infarction event.

FCEs contain ICD-10 codes for a primary diagnosis and up to 19 secondary diagnoses, as detailed in a national-level publication.^12^ We used any mention of myocardial infarction in the first (primary) position of any episode within a spell and used the end date of the most recent myocardial infarction episode to assign the myocardial infarction date. This approach meant that all myocardial infarctions recorded in the primary position were counted, regardless of whether they occurred on admission or during the hospital stay, since all of these myocardial infarction events should be managed in a consistent way using anti-platelet agents and rapid revascularisation where appropriate, followed by secondary prevention therapy.

Consistent with previous studies,^13^ we counted any death within 28 days of a primary myocardial infarction admission as a death following hospitalisation. The HES–ONS linked mortality data capture deaths of people who have been treated in hospitals in England, irrespective of whether they died in hospital or not, and hence include all deaths that were preceded by hospital admission. Using the linked hospitalisation and mortality data, we created three non-overlapping categories of myocardial infarction events: non-fatal events, deaths following hospitalisation for myocardial infarction, and pre-hospital deaths (deaths in patients without a preceding recent hospital admission; appendix pp 6–9). To calculate pre-hospital deaths, we subtracted myocardial infarction deaths following an admission from the total myocardial infarction deaths by age group, sex, and district. Pre-hospital myocardial infarction deaths also include deaths in the ambulance or in the emergency department before formal hospital admission.

We focused on people aged 45 years or older because myocardial infarction is relatively uncommon in younger people and most events—87 966 (98·7%) of 89 124 myocardial infarction-related deaths (ie, deaths with myocardial infarction as the underlying cause, or deaths from other causes within 28 days of a myocardial infarction admission) and 293715 (96·3%) of 305 143 hospitalisations due to myocardial infarction—were in people aged 45 years and older (appendix pp 8–9).

We used data on the Income Deprivation domain of the Index of Multiple Deprivation (the proportion of the population claiming income-related benefits because of being out of work or having low earnings) from the UK Government’s English Indices of Deprivation 2019 statistics^14^ to evaluate inequalities in the components of myocardial infarction mortality among communities of different socioeconomic statuses. The data used for calculating the 2019 indices were sourced from the most recent available timepoint before 2019, which corresponds to our period of analysis. Data were aggregated from lower-layer super output area to district level by population weighting.

### Statistical methods

We did all analyses separately for men and women, and by age group (45–54, 55–64, 65–74, 75–84, and ≥85 years). The number of events or deaths per district, age group, and sex can be small, especially in the younger age groups. Therefore, we used a Bayesian spatial model to obtain stable estimates of non-fatal myocardial infarction events, deaths in patients hospitalised for myocardial infarction, and pre-hospital deaths at the district level. The model, described in the appendix (pp 2–5), is designed to analyse multiple outcomes whose spatial patterns have both similarities and distinct features. Specifically, each outcome has a national intercept, which measures its average level across all districts, and a series of district-specific random intercepts, which measure deviations from the national level. The district-specific random intercepts are specified using two terms: one that is unique to each of the three categories of myocardial infarction events (namely non-fatal myocardial infarction events, deaths in patients hospitalised for myocardial infarction, and pre-hospital deaths) and one that is shared between them. We modelled the shared component with a Besag, York, and Mollie spatial model, which allows the estimates in each district to be influenced by the district’s own data, as well as by those of other districts, especially its neighbours. The extent to which neighbours influence one another depends on how uncertain event rates and mortality are in each district because of small numbers of events, and on the empirical similarity of neighbouring districts. Outcome-specific intercepts for each district were modelled as unstructured random effects. The statistical formulation and reasons for model specification are described in the appendix (pp 2–5).

We used the posterior estimates of non-fatal myocardial infarction events, deaths in patients hospitalised for myocardial infarction, and pre-hospital deaths to calculate the following measures of public health interest for each district (appendix pp 8–9): total event rate (number of myocardial infarction events per 100 000 population), death rate (number of myocardial infarction-related deaths per 100 000 population), total case fatality (proportion of myocardial infarction events that resulted in death), pre-hospital fatality (proportion of myocardial infarction events that resulted in pre-hospital death), and hospital case fatality (proportion of admissions due to myocardial infarction that resulted in death within 28 days). The reported credible intervals (CrIs) represent the 2·5th and 97·5th percentiles of the posterior distributions of each reported metric. We also report the posterior probabilities that the estimated rates are higher or lower than the national average.

We calculated age-standardised death rates and event rates by weighting age-specific rates by the corresponding share of the national population (the denominator used for calculating event and death rates) in each age group. We calculated age-standardised total case fatality and pre-hospital fatality by weighting age-specific case fatality or pre-hospital fatality by the corresponding share of national myocardial infarction events (the denominator used for calculating case fatality) in each age group. We calculated age-standardised hospital case fatality by weighting age-specific hospital case fatality by the corresponding share of national myocardial infarction hospital admissions (the denominator used for calculating hospital case fatality) in each age group.

Myocardial infarction event rate and case fatality act multiplicatively to produce the death rate in each district. As a result, their contributions to variability in death rate are not additive but depend on how much they vary across districts relative to one another and on their correlation. To estimate how much these two components account for the observed variation in death rates, we used a regression analysis. The dependent variable in the regression was district death rate and the sole independent variable was either district event rate or case fatality. We report the share of the total variance of district death rates that is explained separately by event rate and by case fatality, as a measure of their respective contributions to variation in death rate.

We also conducted sensitivity analyses to understand how much our choices about which hospital deaths and which age groups to include would influence our results. In the main analysis, we counted all myocardial infarction deaths without a preceding hospital admission for acute myocardial infarction as pre-hospital deaths, even when they had a preceding non-myocardial infarction admission within 28 days, as has been done in previous studies.^2,13^ In a sensitivity analysis, we counted all acute myocardial infarction deaths within 28 days of any hospital admission—regardless of whether the primary admission diagnosis was myocardial infarction or another condition—as hospital-associated deaths, because these deaths had been recently preceded by an admission and in some cases even occurred while the person was still at the hospital.^12,15^ Only those deaths that had no admission within this window were counted as pre-hospital deaths. We also repeated the analyses with and without inclusion of people aged 85 years and older, because multimorbidity makes the assignment of cause of death or hospitalisation less precise in this age group.

### Role of the funding source

The sponsors of the study had no role in study design, data collection, data analysis, data interpretation, or writing of the report.

## Results

From 2015 to 2018, there were 293 715 myocardial infarction hospitalisations and 87 966 myocardial infarction-related deaths in England among people aged 45 years or older. Ofthese deaths, 76 011 (86·4%) had acute myocardial infarction as the underlying cause of death and the remaining 11 955 (13·6%) were deaths assigned to other causes within 28 days of a myocardial infarction admission (appendix pp 6–9). Of the 76011 deaths with myocardial infarction as the underlying cause, 19 294 (25·4%) occurred within 28 days of a myocardial infarction admission, 20 510 (27·0%) occurred within 28 days of a non-myocardial infarction admission, and 36 207 (47·6%) had no preceding admission. 262 466 (89.3%) of the total myocardial infarction hospitalisations were non-fatal. The national age-standardised myocardial infarction death rate (calculated directly from death and population counts) was 63 per 100 000 population in women and 126 per 100 000 in men, and the national age-standardised event rate was 233 per 100 000 in women and 512 per 100 000 in men. Pre-hospital fatality was 15·0% in women and 16·9% in men, and hospital case fatality was 10·8% in women and 10·6% in men.

**Figure 1:**
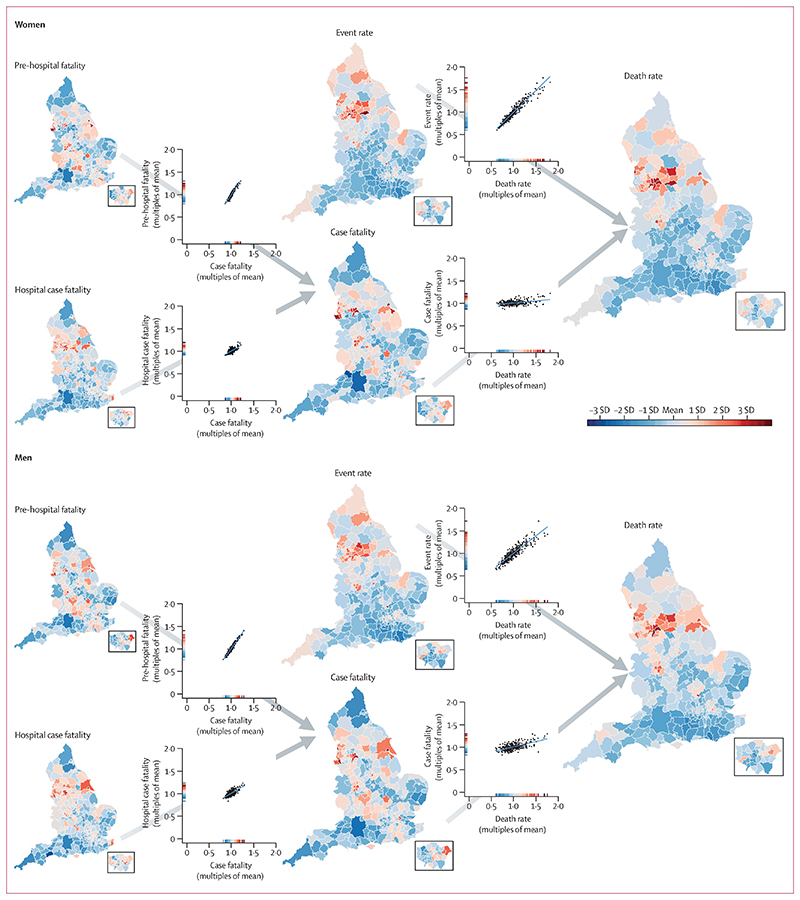
Age-standardised acute myocardial infarction death rate and its contributors in districts of England in women and men The maps show the geography of death rate and each contributor (insets show London). The scatter plots show the relationship between pairs of contributors, or contributors and death rates. All variables were age-standardised. The scale on each scatter plot ranges from 0 to 2 x the mean of the values across all districts to allow the extent of variation to be compared among variables. The colour corresponds to the number of SDs above or below the mean value across all districts. The appendix shows maps and scatter plots with numerical scales (pp 10–11) and the posterior probabilities that the estimated rates and case fatality for each district are higher or lower than the national average (pp 12–13).

The geographical patterns and variations of myocardial infarction mortality and its contributors are shown in figure 1 and in the appendix (pp 10–13). Of contributors to mortality, event rate varied the most, with 99th-to-1st percentile ratios of 2·55 (95% CrI 2·39–2·72) in women and 2·17 (2·08–2·27) in men, compared with 1·36 (1·31–1·43) in women and 1·50 (1·44–1·57) in men for case fatality (table 1). Myocardial infarction mortality was strongly correlated with event rates (correlation coefficients 0·96 for women and 0·91 for men), but only moderately correlated with case fatality (0·39 and 0·61), making event rate the largest driver of the variation in myocardial infarction mortality (99th-to-1st percentile ratio 2·63 [2·45–2·83] in women and 2·56 [2·37–2·76] in men).

**Figure 2:**
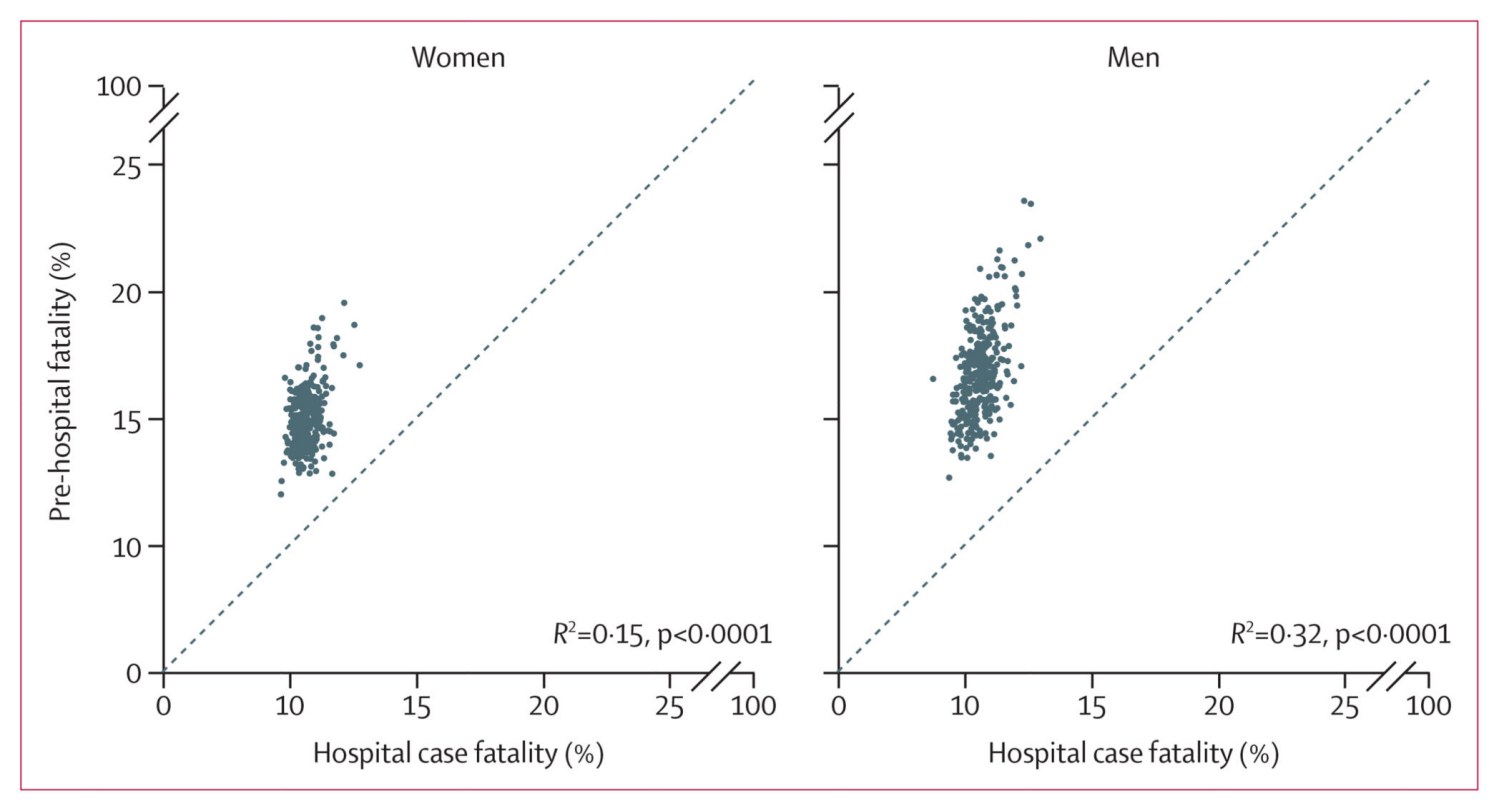
Relationship between pre-hospital fatality and hospital case fatality Each point represents one district.

Variation in total case fatality across districts was driven more by the variation in pre-hospital fatality (99th-to-1st percentile ratio 1·60 [1·50–1·70] in women and 1·75 [1·66–1·86] in men) than hospital case fatality(1·39 [1·29–1·49] and 1·49 [1·39–1·60]). In all 326 districts, pre-hospital fatality was higher than hospital case fatality, by an average factor of 1·40 in women and 1·59 in men (figure 2). Mortality was highest in a cluster of urban districts in the north of England. These districts stood out as having distinctly high myocardial infarction event rates compared with most other districts in England, but were less consistently high with regard to case fatality. For example, Barnsley, Salford, and Luton had higher-than-average event rates in women but lower-than-average case fatality (average refers to the mean across districts for each outcome). Similarly, places such as Luton, Blackburn, Middlesbrough, Hounslow, and County Durham had higher-than-average event rates in men, driving their high death rates, whereas their case fatality was lower than average (figure 1).

**Figure 3:**
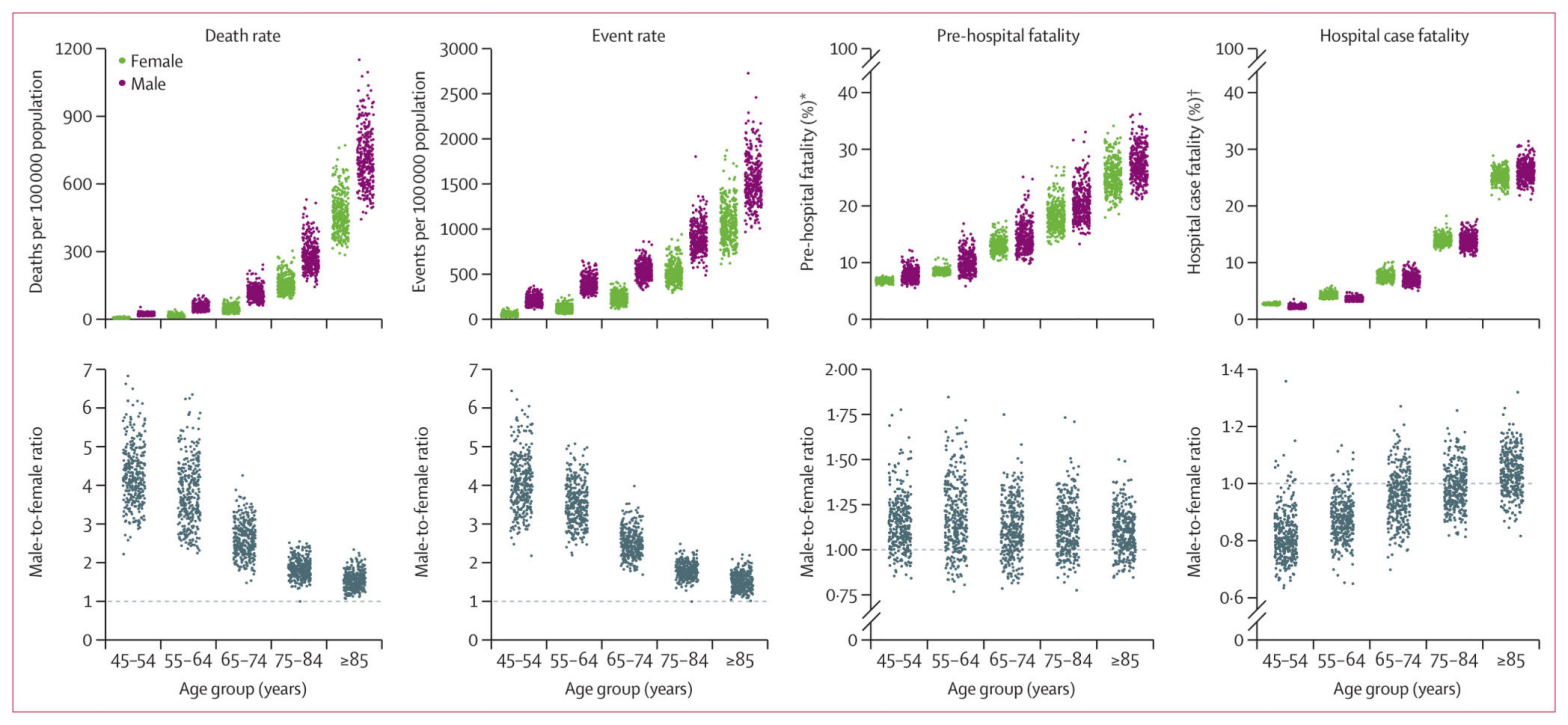
Distribution of myocardial infarction mortality, event rates, pre-hospital fatality, and hospital case fatality by age group and sex, and male-to-female ratios Each point represents one district. *Percentage of all myocardial infarction events. tPercentage of all myocardial infarction hospital admissions.

Myocardial infarction mortality increased by more than 3-fold per decade of age for women and more than 2-fold for men (figure 3). This increase was a result of a rise in both event rates (2·10-fold higher per decade of age for women and 1·61-fold for men) and case fatality, which increased by around 50% per decade of age. The age association of case fatality was more similar to that of pre-hospital fatality (an increase per decade of 1·40-fold in women and 1·38-fold in men) than hospital case fatality (1·75-fold in women and 1·87-fold in men), because the number of pre-hospital deaths exceeded that of deaths following hospitalisation in every age group.

Myocardial infarction death rate was higher in men than in women in every age group and for all districts. Event rate was also consistently higher in men than in women, in all except two districts in the oldest age groups (75–84 and ≥85 years). In some districts, death and event rates in men exceeded those in women by more than five times in the 45–54 and 55–64 years age groups, and up to four times in those aged 65–74 years. Pre-hospital fatality was slightly higher in men than in women in most districts and age groups, whereas hospital case fatality was higher in women in almost all districts in the 45–54 years and 55–64 years age groups; in higher age groups, it was more similar between the sexes (figure 3).

In all age groups, variation in event rates made a greater contribution to how much myocardial infarction mortality varied across districts than did variation in case fatality (table 2). The relative importance of case fatality was highest in women aged 55–64 and 65–74 years and in men aged 55–64, 65–74, and 75–84 years, and was lowest in the youngest age groups (in which case fatality is low in all districts) and the oldest age groups (in which case fatality is high in all districts).

**Figure 4:**
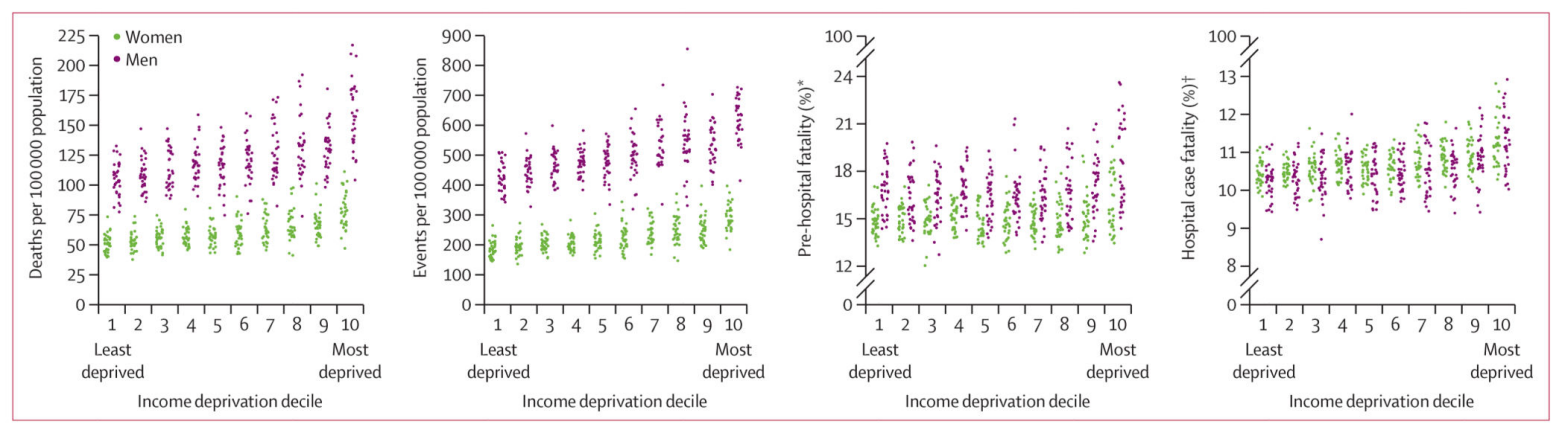
Distribution of myocardial infarction mortality, event rates, pre-hospital fatality, and hospital case fatality by decile of income deprivation Each point represents one district. *Percentage of all myocardial infarction events. tPercentage of all myocardial infarction hospital admissions.

Myocardial infarction death rate and event rate were directly associated with district-level income deprivation (figure 4). For pre-hospital fatality and hospital case fatality, the distributions were similar for most deciles, except for the poorest 20% of districts. In these two deciles, higher proportions of myocardial infarction events led to death before reaching a hospital and higher proportions of hospitalised patients died than in the other districts. The variation in death rates, event rates, and fatality were all larger within each decile of income deprivation than across the deciles.

Sensitivity analyses showed that inclusion of all acute myocardial infarction deaths within 28 days of any admission (regardless of whether the primary admission diagnosis was myocardial infarction or another condition; an additional 25 510 deaths) as post-hospitalisation deaths increased hospital case fatality by 4·7–8·2 percentage points and decreased pre-hospital fatality by 4·4–8·2 percentage points across different districts and the two sexes. As a result, the degree of variation in pre-hospital fatality among districts increased, but the overall ranking of districts in terms of high versus low hospital case fatality and pre-hospital fatality was maintained; the correlation coefficients between the results of the main and sensitivity analyses were 0·93 (women) and 0·95 (men) for district-level hospital case fatality, and 0·91 (women) and 0·94 (men) for district-level pre-hospital fatality.

All outcomes were correlated between the analyses done with and without including people aged 85 years and older. Correlation coefficients between the two age groups (≥45 years and 45–85 years) ranged from 0·96 to 0·99 for death rates and event rates in the two sexes, and from 0·89 to 0·96 for total case fatality, pre-hospital fatality, and hospital case fatality, possibly because case fatality is much higher in the oldest ages (figure 3).

## Discussion

We found that variation in hospital case fatality made only a small contribution to the substantial geographical variation in myocardial infarction mortality in England from 2015 to 2018. A much bigger element of this variation in mortality arose from pre-hospital deaths and event rates. Hospital case fatality, nonetheless, varied across districts.

Our results are based on nationwide linked data that capture all forms of myocardial infarction: non-fatal, and pre-hospital and post-admission fatal. These distinctions are key to designing and evaluating interventions that target the most important deter-minants of mortality. The use of routine health-care data, while enabling a national analysis, has some limitations. ICD-10 codes summarise diagnoses but do not specify clinical investigations and laboratory results. Thus, ST-elevation myocardial infarction (STEMI) and non-STEMI cannot be definitively distinguished. The assignment of ICD-10 codes might also vary across physicians and hospitals. Nonetheless, the quality and consistency of coding of myocardial infarction in routine hospital data have been evaluated against clinical disease registries and chart reviews of myocardial infarction using established diagnostic criteria, with sensitivity and positive predictive values reported to be 79–95%, and hospitalisation data having higher validity than mortality statistics.^16,17^ We did not include cases of myocardial infarction diagnosed as a secondary condition because the recording of secondary diagnoses is more variable than that of primary diagnoses, and the causes of secondary myocardial infarction, as well as the treatment pathways, might differ. Nationally, the inclusion of cases of myocardial infarction recorded as a secondary diagnosis would lead to around a 37% increase in total myocardial infarction admissions.^12^ Cause-of-death assignment is based on more limited clinical information than hospital diagnostic codes—eg, in cases of pre-hospital cardiac arrest—and thus might be more subject to error.^17^ It is unlikely, however, that cause-of-death assignment varies subnationally enough to affect the results.

To our knowledge, no previous study has analysed small-area variation in myocardial infarction deaths and its constituents (non-fatal events and pre-hospital and post-admission deaths). Some studies have reported national data for specific countries; most of these studies relied on data from hospitalised patients (ie, excluding pre-hospital deaths),^18–27^ and only some included pre-hospital deaths.^1,2,28–31^ Our estimated hospital case fatality of around 11% is consistent with the national audit data report in England,^32^ and within the 4–17% range in member countries of the Organisation for Economic Co-operation and Development.^33^ Because few studies used data on pre-hospital deaths,^1,2,28–31^ there is little comparative data on total case fatality and especially on the percentage of events that lead to death before hospitalisation. Consistent with our finding across districts, these studies found that pre-hospital fatality was a larger contributor to case fatality than was hospital case fatality. Pre-hospital fatality also varied more across countries than did hospital case fatality.

The current standard of care for myocardial infarction in England and other nations is resuscitation, early diagnosis with electrocardiography, and rapid transfer of patients with STEMI to a percutaneous coronary intervention centre for immediate revascularisation. Reorganisation of emergency services to facilitate rapid transfer, reduction in door-to-balloon reperfusion times, and universal anti-platelet therapy are among the reasons for hospital case fatality having the least variation among the constituents of myocardial infarction mortality, both within England and across high-income countries. The remaining variations in hospital case fatality might be partly due to differences in reperfusion times and percutaneous coronary intervention capacity, adherence to guidelines, or patient comorbidities.^23,34,35^ Management of non-STEMI, which relies on risk stratification to decide on early versus delayed angiography and on optimal anti-coagulant and anti-platelet therapy, also accounts for some of the observed variations in hospital case fatality.^34–37^ Finally, the use of secondary prevention therapies in the immediate post-myocardial infarction phase, which improves both 28-day and longer-term survival, also varies within England and across countries.^35,37^

As hospital case fatality has declined and become less variable, pre-hospital fatality plays a larger relative role in the survival of patients and its variations across and within countries. Important determinants of pre-hospital fatality include the time taken to recognise symptoms and call for and receive help, and the use of pre-hospital cardiopulmonary resuscitation (CPR) and pre-hospital defibrillation in the event of cardiac arrest.^38,39^ England and other high-income countries have implemented awareness campaigns for myocardial infarction symptoms,^40^ but these programmes are rarely targeted and adapted to communities where pre-hospital deaths are high. Strategies to reduce mortality from cardiac arrest following myocardial infarction^38,39^ include increasing CPR competence in the general public (Japan and Scotland),^41,42^ with support by emergency services via telephone (New Zealand and Singapore);^43,44^ using trained volunteer or fire, police, or health-service workers as first responders (Austria, Norway, and Ireland);^45^ increasing the number of public-access defibrillators;^46^ and alerting nearby CPR-trained responders using mobile phone alerts (Denmark and England).^47,48^ The available data show that some of the potentially effective interventions, such as public-access defibrillators, are used less commonly than standardised facility-level interventions; the use of other interventions, such as bystander CPR, varies across and within countries.^49^

Alongside lowering case fatality where it remains high, our results show that there is a need and potential to further reduce myocardial infarction event rates in many parts of England because the sheer size of case numbers can drive areas that benefit from low case fatality into high mortality rankings, and vice versa. Event rates are influenced by smoking and risk factors such as blood pressure, lipid levels, diabetes, and obesity, which mediate the effects of nutrition and the environment. In England, these risk factors tend to be higher where myocardial infarction event rates are highest.^50–52^ These risks can be partly reduced through more ambitious and equitable preventive interventions, such as New Zealand’s recent zero-smoking policy and financial support for healthy foods.^53^ Risk can also be effectively mitigated by individual-level primary and secondary prevention through counselling for smoking cessation, statin therapy, and treatment of hypertension and diabetes. In England, cardiovascular risk screening has been offered to approximately 33% of the eligible population, of whom only about 50% take it up, leaving many of those at risk unscreened and untreated; the extent of undertreatment varies across the country.^54,55^

The decline in myocardial infarction mortality over the past five decades, driven by lower levels of smoking and other risk factors and advances in treatment both in primary care and specialist hospitals, has been a major clinical and public health success in high-income nations. Hospital case fatality is the element of the acute myocardial infarction pathway that is most relevant to those myocardial infarction patients who reach a facility, and most amenable to direct health-system intervention. However, with standardisation of hospital care following randomised trials, hospital case fatality now makes a smaller contribution to variations in myocardial infarction mortality within England and across high-income nations than do pre-hospital deaths and event rates. Nonetheless, the combination of our results and data on cross-country variations in hospital fatality show that further improvement in England is possible but requires a subnational focus where hospital case fatality remains high.

Our results also show that further scaling up population-based and individual-level primary and secondary prevention, as well as addressing the relatively large and highly variable pre-hospital fatality, are essential to reducing overall mortality. Strategies to achieve these reductions should be evaluated in randomised trials and in real-world conditions when new programmes are implemented. To ensure that these interventions translate to beneficial impact on death rates, there should be focus on parts of the country where each constituent of mortality is highest, and enhancement of registries to gather data on deaths outside the hospital setting, as currently done for hospitalised patients.

## Supplementary Material

Supplementary Appendix

## Figures and Tables

**Table 1:** Distributions of myocardial infarction mortality and its components (event rate and case fatality, including pre-hospital fatality and hospital case fatality) across 326 districts in England

	Mortality (per 100 000 population)	Event rate (per 100 000 population)	Total case fatality (%)	Pre-hospital fatality* (%)	Hospital case fatality^†^ (%)
**Women**					
Best performing district	38 (33–44)	136 (124–149)	20·8% (19·0–22·6)	12·0% (10·5–13·7)	9·7% (8·6–10·8)
1st percentile	41 (36–47)	148 (131–166)	22·1% (20·4–23·9)	12·9% (11·3–14·5)	9·9% (8·7–11·1)
25th percentile	52 (45–60)	193 (177–210)	23·3% (21·1–25·6)	14·2% (12·3–16·4)	10·4% (9·3–11·6)
50th percentile	59 (52–68)	218 (196–241)	24·0% (21·8–26·3)	14·9% (13·3–16·6)	10·7% (9·4–12·0)
75th percentile	68 (59–77)	252 (222–285)	24·7% (22·6–26·9)	15·7% (13·6–18·2)	10·9% (9·7–12·3)
99th percentile	102 (91–113)	370 (341–400)	27·9% (25·8–30·0)	18·6% (16·7–20·6)	12·2% (10·9–13·5)
Worst performing district	111 (99–125)	397 (374–421)	29·2% (27·0–31·5)	19·6% (17·4–21·8)	12·8% (11·5–14·2)
Ratio of 99th to 1st percentile	2·63 (2·45–2·83)	2·55 (2·39–2·72)	1·36 (1·31–1·43)	1·60 (1·50–1·70)	1·39 (1·29–1·49)
**Men**					
Best performing district	74 (63–86)	319 (293–348)	20·9% (19·5–22·4)	12·7% (11·5–14·0)	8·7% (7·6–9·8)
1st percentile	78 (67–90)	335 (305–364)	22·2% (20·0–24·4)	13·6% (12·2–15·0)	9·4% (8·5–10·3)
25th percentile	106 (95–118)	444 (408–482)	24·2% (21·9–26·6)	15·5% (13·4–17·9)	10·1% (8·8–11·5)
50th percentile	119 (106–133)	492 (456–529)	25·4% (22·9–27·9)	16·6% (15·1–18·2)	10·5% (9·6–11·4)
75th percentile	134 (119–150)	548 (512–585)	26·5% (24·4–28·6)	17·7% (15·2–20·4)	10·9% (9·6–12·4)
99th percentile	192 (172–213)	721 (689–754)	31·6% (29·5–33·7)	21·9% (19·8–23·9)	12·3% (11·0–13·6)
Worst performing district	217 (195–240)	855 (807–904)	32·9% (30·5–35·3)	23·6% (21·9–25·3)	12·9% (11·4–14·6)
Ratio of 99th to 1st percentile	2·56 (2·37–2·76)	2·17 (2·08–2·27)	1·50 (1·44–1·57)	1·75 (1·66–1·86)	1·49 (1·39–1·60)

Mortality, event rate, and fatality all apply to myocardial infarction. Numbers in parentheses are 95% credible intervals. The best performing and worst performing districts (ie, the individual districts with the lowest and highest values, respectively) could be different for each outcome. *Proportion of myocardial infarction events that result in death before hospital admission. †Proportion of hospital admissions due to myocardial infarction that result in death within 28 days of admission.

**Table 2:** Proportion of variation in myocardial infarction mortality across districts explained by myocardial infarction event rates and case fatality, by sex and age group

	Proportion of variation explained by event rate	Proportion of variation explained by case fatality
**Women**		
45–54 years	98·8%	21·5%
55–64 years	98·7%	71·1%
65–74 years	93·9%	50·6%
75–84 years	86·4%	20·8%
≥85 years	91·3%	<0·1%
**Men**		
45–54 years	81·4%	7·8%
55–64 years	74·3%	47·8%
65–74 years	71·0%	62·7%
75–84 years	78·6%	47·1%
≥85 years	86·2%	7·7%

Percentages show how much less variable myocardial infarction death rates would be if that contributor (event rate or case fatality) was at the same level in all districts in that age-sex group. Myocardial infarction event rates and case fatality act in a multiplicative manner in each district to produce the death rate and are not independent; thus, the contributions do not add to 100%.

## Data Availability

The Small Area Health Statistics Unit does not have permission to release data to third parties except in the form of non-disclosive statistical tables or conclusions suitable for publication. Individual mortality data can be requested through the ONS website. HES data can be requested through NHS Digital.

## Research in context

### Evidence before this study

We searched MEDLINE (via PubMed) for articles published from Jan 1, 2000, to Dec 6, 2021, using the search terms (“myocardial infarction”[Title] OR “coronary heart disease”[Title] or “ischaemic heart disease”[Title]) AND ((“subnational”) OR (“small area”) OR (“local”)) AND ((“registry”) OR (“incidence”) OR (“mortality”) OR (“case fatality”)). No language restrictions were applied. We started our search from the year 2000 in order to focus on studies after the introduction of primary angioplasty and the use of troponin-based measurements to define myocardial infarction. We also searched for relevant reports through the websites of registries and requests for information from clinicians and researchers in high-income countries in Australasia, Europe, and the Americas. We found some studies from countries in Australasia, Europe, and the Americas that had used data on hospitalised patients and reported myocardial infarction admissions and hospital case fatality for an entire country. The Organisation for Economic Co-operation and Development also reports hospital case fatality for its member states based on countries’ officially reported statistics, but the actual data sources are not stated. Few of these studies included deaths outside of a hospital setting; of these, some had considered all ischaemic heart disease deaths, and only four national studies had specifically focused on pre-hospital acute myocardial infarction deaths.

We also found a study that used data from specific communities in six countries to report on myocardial infarction admissions and pre-hospital and hospital case fatality, as had been done in the MONICA study for the 1990s. In terms of subnational studies, we found one study on death rates for ischaemic heart disease for US counties, and two reports of ischaemic heart disease death rates for local authorities in England. These studies did not separate pre-hospital versus hospital fatality or distinguish acute myocardial infarction from chronic atherosclerotic disease and complications; nor did they have data on hospitalisation. We also found one local authority-level study of myocardial infarction hospitalisation rates in England, but this study did not include pre-hospital deaths. To our knowledge, there is no study on subnational variations in myocardial infarction death rate and its complete contributors (event rates, pre-hospital fatality, and hospital case fatality).

### Added value of this study

To our knowledge, this study provides the only subnational analysis of myocardial infarction death rate and its complete contributors in any country. We used nationwide linked data that capture all forms of myocardial infarction events: non-fatal events and pre-hospital and hospital fatality. We used a spatial statistical model to obtain stable estimates of myocardial infarction event rates and pre-hospital fatality and hospital case fatality by age group for small geographies, together with the uncertainty in these estimates.

### Implications of all the available evidence

Our subnational results, together with available national data, show that pre-hospital deaths are a larger contributor to myocardial infarction mortality, and how it varies both within and across high-income countries, than is hospital case fatality. This finding demonstrates the need for research on, and implementation and standardisation of, interventions that reduce time between symptom onset and call for help, as well as the time to initiation of cardiopulmonary resuscitation and defibrillation in the event of a myocardial infarction leading to cardiac arrest. There is also a need for regular national and subnational reporting of all myocardial infarction deaths, separated by whether the individual had a recent hospital admission, so that the impacts of interventions can be measured.
