# Supplementary material for: Contributions of event rates, pre-hospital deaths, and deaths following hospitalisation to variations in myocardial infarction mortality in 326 districts in England: a spatial analysis of linked hospitalisation and mortality data
Source: Lancet Public Health. Author manuscript; Available in PMC 2023 Sep 23. (PMC10506182; doi:10.1016/S2468-2667(22)00108-6)
Supplement: Supplementary Appendix [file EMS188113-supplement-Supplementary_Appendix.pdf]

THE LANCET
Public Health

Supplementary appendix

This appendix formed part of the original submission and has been peer reviewed. We post it as supplied by the authors.

Supplement to: Asaria P, Bennett JE, Elliott P, et al. Contributions of event rates, pre-hospital deaths, and deaths following hospitalisation to variations in myocardial infarction mortality in 326 districts in England: a spatial analysis of linked hospitalisation and mortality data. *Lancet Public Health* 2022; published online July 15. https://doi.org/10.1016/S2468-2667(22)00108-6.

Table of contents

Appendix Text 1: Specification and implementation of the Bayesian statistical model 2

Appendix Figure 1: Flowchart for the use of data on hospital admissions and deaths. 6

Appendix Figure 2: Schematic diagram showing the division of myocardial infarction (MI) into pre-hospital deaths, hospital deaths and non-fatal events, and the two sources of data used for their calculation.. 8

Appendix Figure 3: Age-standardised myocardial infarction (MI) death rates and its contributors in English districts. 10

Appendix Figure 4: Posterior probability (pp) maps for age-standardised myocardial infarction (MI) death rates and its contributors in English districts. 12

Appendix Figure 5: Post burn-in and thinning trace plots of key parameters and examples of district specific rates. 14

References 25

Appendix Text 1: Specification and implementation of the Bayesian statistical model

As stated in the main paper, we used a Bayesian spatial model to obtain stable estimates of non-fatal MI events and pre-hospital and post admission fatal events. The model is designed to analyse multiple outcomes whose spatial patterns have both similarities and distinct features. Models were run separately for men and women and by age group. For each outcome $j\ (= 1, 2,3)$, the number of events or deaths in a district $d\ (= 1, \dots, 326)$ follows a Poisson distribution:

$$y_{jd} \sim \text{Poisson}\left(\lambda_{jd} \,.population_d\right),$$

where the coefficients λ_{jd} are rates in the district population. The log-transformed rates are modelled as a sum of intercept and district specific terms:

$$\log\left(\lambda_{jd}\right) = \alpha_j + \eta_{jd}.$$

The α_j are common intercepts for each outcome. The terms η_{jd} are specified as a sum of shared and outcome specific random effects,

$$\eta_{jd} = \varphi_d . \delta_j + \psi_{jd}.$$

The φ_d are shared random effects common to all three outcomes and are modelled following the Besag, York and Mollie (BYM) model[1] and the ψ_{jd} are spatially-unstructured (independent and identically distributed, IID) random effects for each of the three outcomes. δ_j is a scaling parameter which determines the size of the contribution of the shared component φ_d to the overall deviation from the common level for outcome j.

The BYM specification models these random effects as the combination of spatially-structured random effects with intrinsic conditional autoregressive (ICAR) priors, allowing information to be shared locally between neighbouring districts, and spatially-unstructured IID random effects, allowing information to be shared amongst all districts. We implement this using the BYM2 parameterisation[2] with

$$\varphi_d = \sigma_\varphi . (V_d\sqrt{1-\rho} + U_d\sqrt{\frac{\rho}{SF}})$$

$$V_d \sim N[0, 1]$$

$$U_d \sim ICAR[W, 1]$$

where σ_φ is the overall standard deviation of the shared random effects. V_d are the unstructured shared random effects. U_d are the structured shared random effects arising from an ICAR with standard deviation set to 1 and adjacency matrix W. The conditional distribution for U_d, with ω_d the set of neighbours of district d and $n_d$the number of districts in ω_d, can be written as

$$U_d | U_{-d} \sim N\left[\frac{1}{n_d}\sum_{k\in\omega_d} U_k, \frac{1}{n_d}\right].$$

The scaling factor *SF* in the BYM2 equation ensures that $Var(U_d) \approx 1$ and is calculated from the adjacency matrix. Finally, the mixing factor ρ determines how much of the variance comes from the unstructured versus the structured effects.

Neighbours of districts are typically determined by shared land borders, which do not exist for islands, necessitating study-specific choices about neighbourhood.[3] For our analysis, the districts of the Isle of Wight and the Isles of Scilly were each joined to the nearest mainland district based on ferry connections, following other analyses in England[4,5] and similar analyses in the USA.[6]

For the scaling parameters $\delta_{j=1,2}$ we assigned the prior $\log(\delta_j) \sim N[0, \frac{1}{5.9}]$, in which 95% of the probability of the prior for ${\delta_j}^2$ lies between 0.2 and 5.[7] So that the sum to zero constraint is maintained, we set $\log(\delta_3) = -\log(\delta_1) - \log(\delta_2)$.

Weakly informative or flat priors were used so that inference on the parameters was driven by the data. The common intercepts were assigned diffuse priors. A half-normal distribution with unit variance was assigned to each of the precision parameters of the district specific random effects. And the mixing parameter ρ was given a flat prior via a $Beta[1,1]$ distribution.

$$\alpha_{j,j=1,2,3} \sim N(0, 10000)$$

$$\sigma_{\psi_{j,j=1,2,3}} \sim \ half\text{-}N(0, 1)$$

$$\sigma_{\varphi} \sim half\text{-}N(0, 1)$$

$$\rho \sim Beta[1,1]$$

We considered alternative formulations of the shared and outcome specific components as some previous analyses have used the same CAR specification or spatial cluster model for the shared as well as disease specific components[7,8] while others have used CAR for the shared components and non-spatial distributions for the disease specific components.[9-11] As detailed above, we used a BYM model for the shared component and IID random effects for the outcome specific components. We did not use four spatially structured (e.g., CAR) terms for both epidemiological and statistical reasons: Epidemiologically, although event rate as a whole may have a spatial structure – which led to the decision to use a BYM formulation for the shared component – we did not expect factors that affect either pre-hospital deaths (e.g., time to recognise symptoms and A&E response) or hospital performance in one district to be any more similar to a neighbouring district than to one further away. This expectation was confirmed by our posterior results. Therefore, using a spatially structured prior for each of these individual quantities would risk smoothing away the very variations that we seek to uncover. Statistically, we tried multiple permutations of the model, which we assessed using DIC. The DIC values for the model using four CAR priors was greater than the DIC for the model used in the paper.

Implementation

Inference was performed using Markov chain Monte Carlo via NIMBLE.[12,13] We monitored convergence using trace plots and the Brooks, Gelman Rubin diagnostic,[14] and thinned post burn-in samples to reduce memory and storage use. We ran two chains for 2,520,000 iterations, discarding the first 20,000 and thinning the remainder by 500 to obtain 10,000 post-

burn-in draws from the posterior distribution of model parameters. Trace plots for key parameters as well for 2 example districts are shown in Appendix Figure 5.

Sensitivity analysis

In sensitivity analysis performed in WinBUGS[15] we repeated the analysis using the standard parameterisation of BYM with *gamma(0.5, 0.0005)* priors assigned to each of the precision parameters of the district specific random effects. Correlations between the four sets of age-standardised estimated rates (event rate, mortality, pre-hospital case fatality and hospital case fatality) in the sensitivity and main analyses were greater than 0.99.

Appendix Figure 1: Flowchart for the use of data on hospital admissions and deaths.

Data only include people aged 45 years and older. Deaths and admissions were from 01/01/2015 to 31/12/2018. Admissions linked to death from acute myocardial infarction within this period might have occurred up to 28 days before the start of this period and were included. Deaths linked to admissions within this period might have occurred up to 28 days after the end of the period and were included. We used a continuous inpatient spell algorithm to collapse finished consultant episodes into admissions and then collapsed them further so that admissions within 28 days of the index event were counted as part of the same event. A Venn diagram representation of the data is available in Appendix Figure 2.

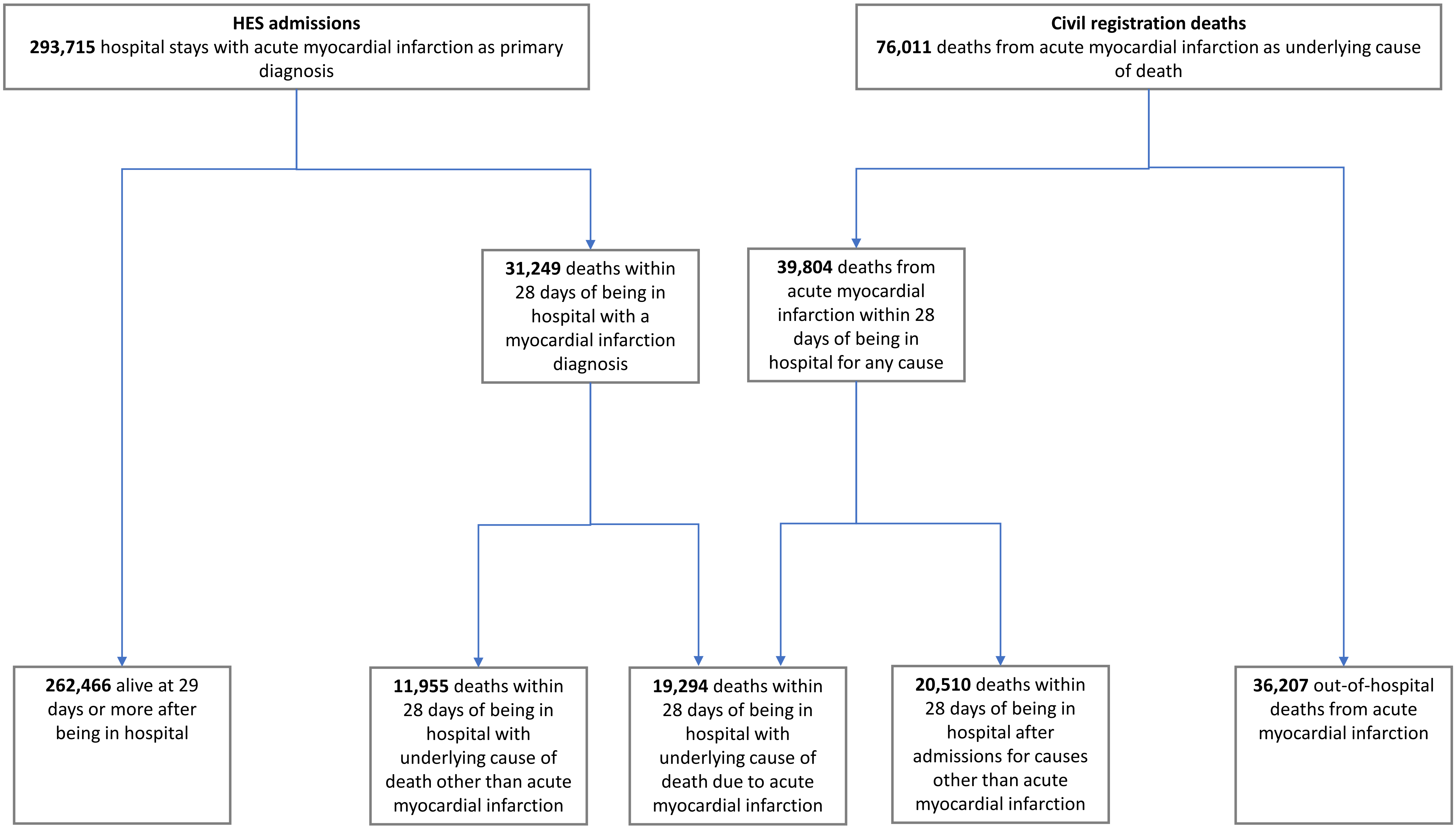
HES admissions
293,715 hospital stays with acute myocardial infarction as primary diagnosis
Civil registration deaths
76,011 deaths from acute myocardial infarction as underlying cause of death
31,249 deaths within 28 days of being in hospital with a myocardial infarction diagnosis
39,804 deaths from acute myocardial infarction within 28 days of being in hospital for any cause
262,466 alive at 29 days or more after being in hospital
11,955 deaths within 28 days of being in hospital with underlying cause of death other than acute myocardial infarction
19,294 deaths within 28 days of being in hospital with underlying cause of death due to acute myocardial infarction
20,510 deaths within 28 days of being in hospital after admissions for causes other than acute myocardial infarction
36,207 out-of-hospital deaths from acute myocardial infarction

Appendix Figure 2: Schematic diagram showing the division of myocardial infarction (MI) into pre-hospital deaths, hospital deaths and non-fatal events, and the two sources of data used for their calculation. All numbers are for people aged 45 years and older.

A: non-fatal events

B (B1+B2): hospital deaths

C (C1+C2): pre-hospital deaths in the main analysis; in the sensitivity analysis, C1 was counted towards hospital deaths as described in Methods

Total number of events = A+B+C

Total event rate = (A+B+C) / population

Death rate = (B+C) / population

Total case fatality = (B+C) / (A+B+C)

Pre-hospital fatality (share of events which result out-of-hospital death) = C / (A+B+C)

Hospital case fatality = B / (A+B)

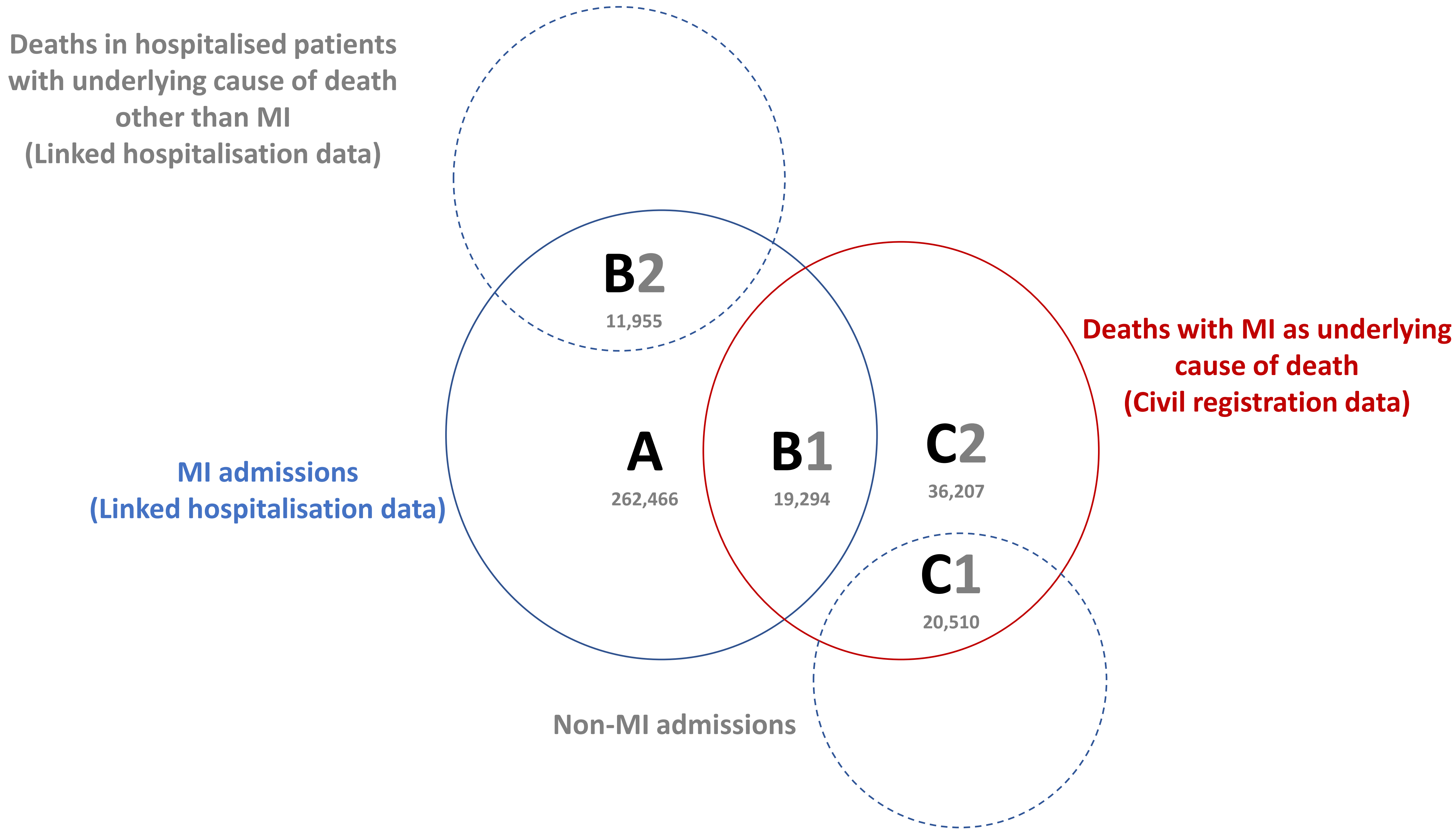
Deaths in hospitalised patients with underlying cause of death other than MI (Linked hospitalisation data)
B2
11,955
MI admissions (Linked hospitalisation data)
A
262,466
B1
19,294
C2
36,207
Deaths with MI as underlying cause of death (Civil registration data)
C1
20,510
Non-MI admissions

Appendix Figure 3: Age-standardised myocardial infarction (MI) death rates and its contributors in English districts.

The maps show the geography of death rate and each contributor with absolute numerical scales. The insets show London. The scatter plots show the relationship between pairs of contributors, or contributors and death rates. All variables were age-standardised as described in Methods. The scale on each scatter plot ranges from 0 to 2 x mean value, so that the extent of variation can be compared among variables. The colour is determined by the number of standard deviations above or below the mean value across all districts.

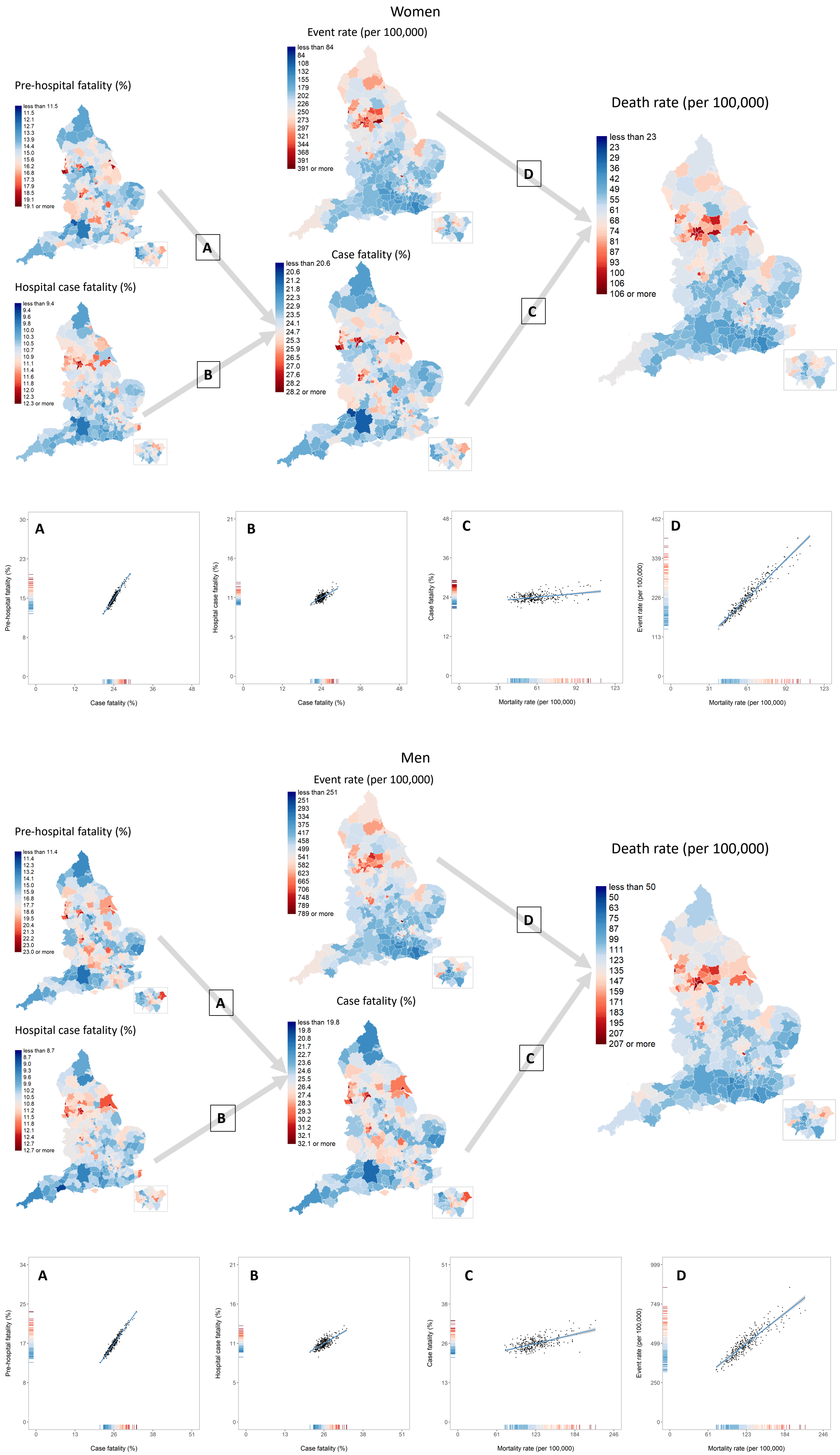

Women
Event rate (per 100,000)
less than 84
84
108
132
155
179
202
226
250
273
297
321
344
368
391
391 or more
Pre-hospital fatality (%)
less than 11.5
11.5
12.1
12.7
13.3
13.9
14.4
15.0
15.6
16.2
16.8
17.3
17.9
18.5
19.1
19.1 or more
Death rate (per 100,000)
less than 23
23
29
36
42
49
55
61
68
74
81
87
93
100
106
106 or more
Case fatality (%)
less than 20.6
20.6
21.2
21.8
22.3
22.9
23.5
24.1
24.7
25.3
25.9
26.5
27.0
27.6
28.2
28.2 or more
Hospital case fatality (%)
less than 9.4
9.4
9.6
9.8
10.0
10.3
10.5
10.7
10.9
11.1
11.4
11.6
11.8
12.0
12.3
12.3 or more
A
B
C
D
Pre-hospital fatality (%)
Case fatality (%)
Hospital case fatality (%)
Case fatality (%)
Mortality rate (per 100,000)
Event rate (per 100,000)
Mortality rate (per 100,000)
Men
Event rate (per 100,000)
less than 251
251
293
334
375
417
458
499
541
582
623
665
706
748
789
789 or more
Pre-hospital fatality (%)
less than 11.4
11.4
12.3
13.2
14.1
15.0
15.9
16.8
17.7
18.6
19.5
20.4
21.3
22.2
23.0
23.0 or more
Death rate (per 100,000)
less than 50
50
63
75
87
99
111
123
135
147
159
171
183
195
207
207 or more
Case fatality (%)
less than 19.8
19.8
20.8
21.7
22.7
23.6
24.6
25.5
26.4
27.4
28.3
29.3
30.2
31.2
32.1
32.1 or more
Hospital case fatality (%)
less than 8.7
8.7
9.0
9.3
9.6
9.9
10.2
10.5
10.8
11.2
11.5
11.8
12.1
12.4
12.7
12.7 or more

Appendix Figure 4: Posterior probability (pp) maps for age-standardised myocardial infarction (MI) death rates and its contributors in English districts.

The insets show London. Posterior probability represents the uncertainty in the district-level value for each outcome. In a district in which the event rate, mortality or case-fatality is the same as the posterior median national value, there is a 50% posterior probability that the district rate is greater than the national rate and a 50% posterior probability that the district rate is less than the national rate. Posterior probabilities more distant from 50%, toward either 0% or 100%, indicate more certainty. Where the entire posterior distribution of the district rate is smaller than the national median, there is a ~100% posterior probability of a higher rate and a ~0% posterior probability of a lower rate, and vice versa. For most districts, the posterior distribution of event rates, case fatality and mortality covers the national rate, with the two parts of the distribution summing to 100% (for example, 80% posterior probability of a higher than national rate and 20% of a lower rate).

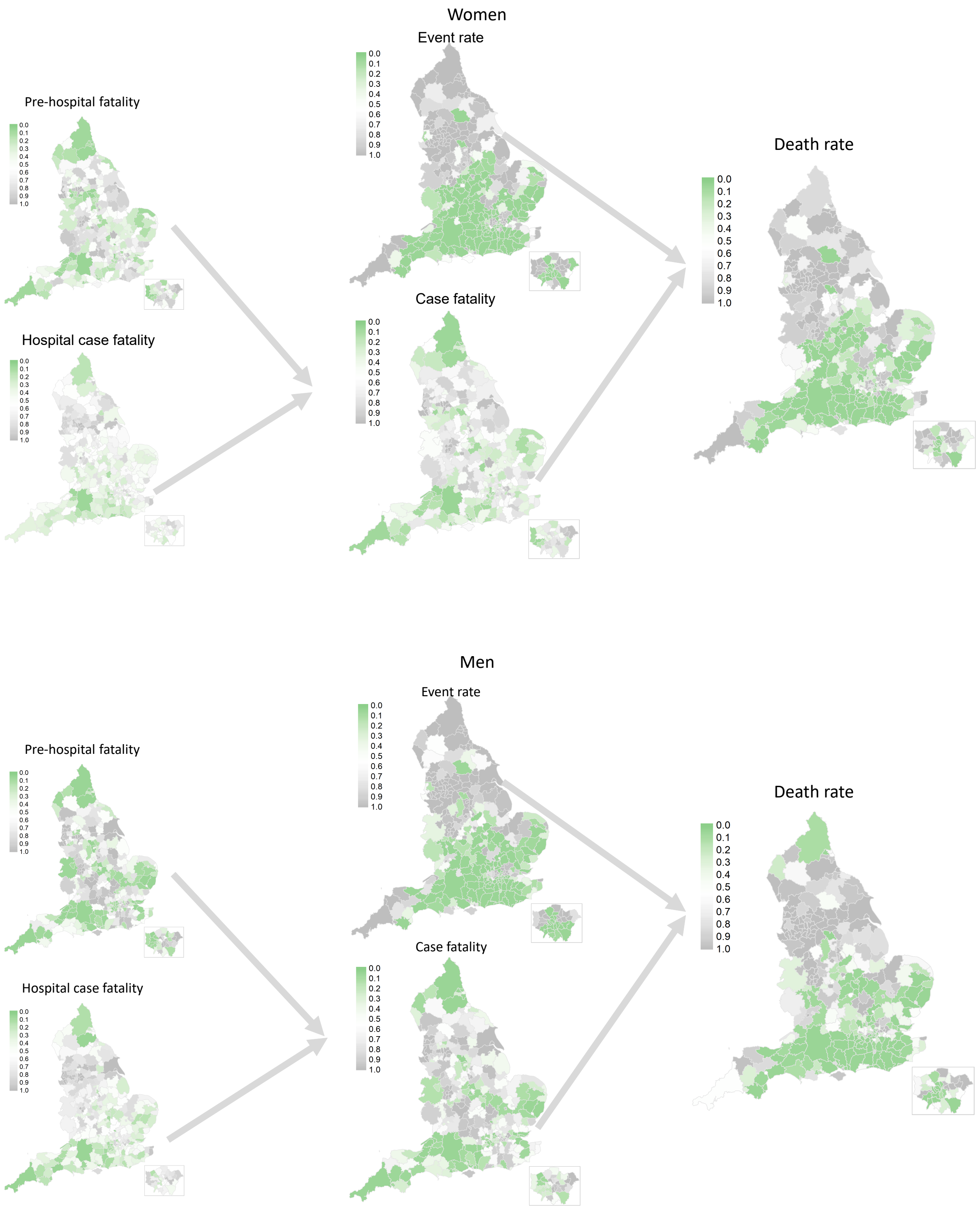
Women
Pre-hospital fatality
Hospital case fatality
Event rate
Case fatality
Death rate
0.0
0.1
0.2
0.3
0.4
0.5
0.6
0.7
0.8
0.9
1.0
Men
Pre-hospital fatality
Hospital case fatality
Event rate
Case fatality
Death rate

Appendix Figure 5: Post burn-in and thinning trace plots of key parameters and examples of district specific rates.

Traceplots women aged 45−54 years

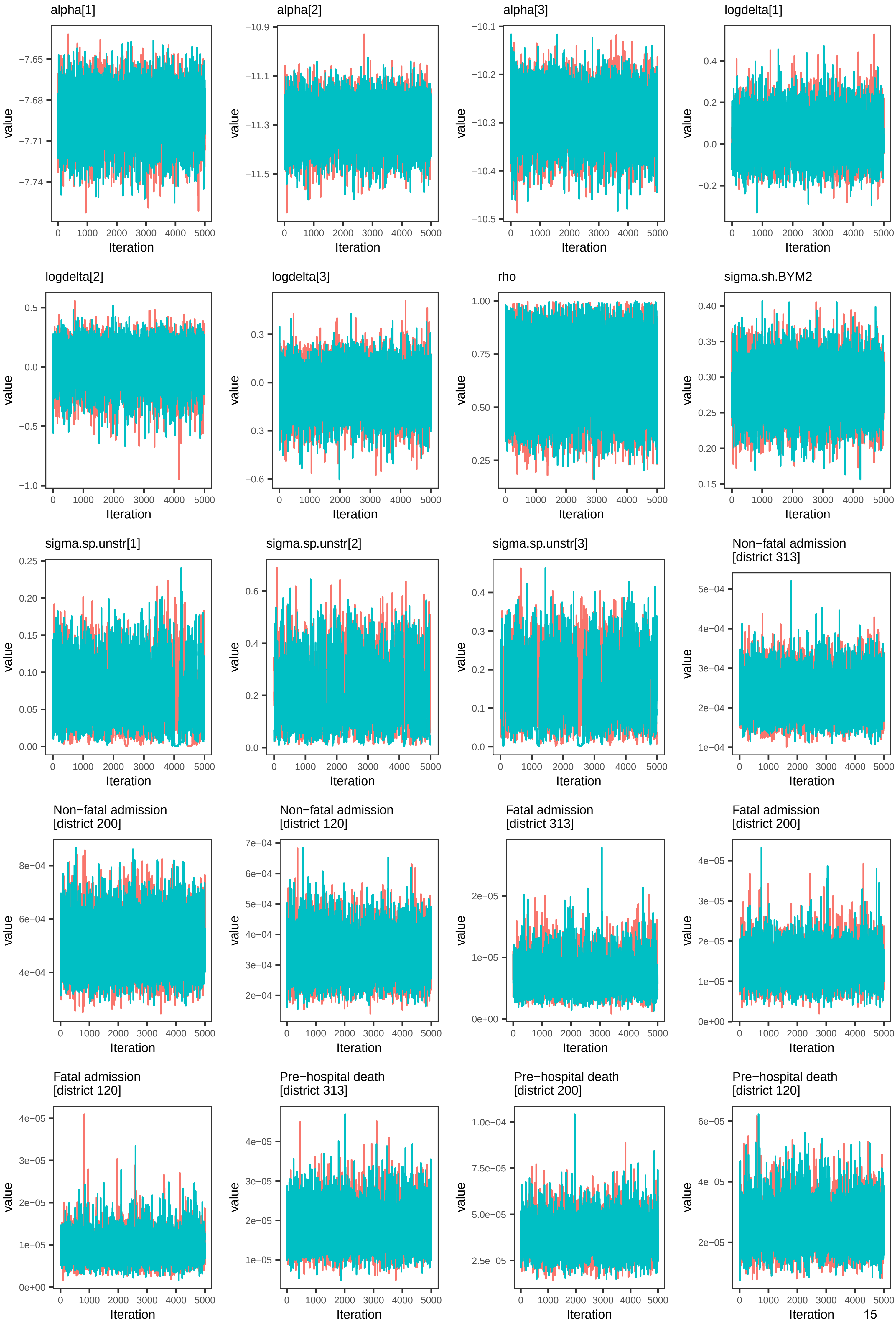

Traceplots women aged 55−64 years

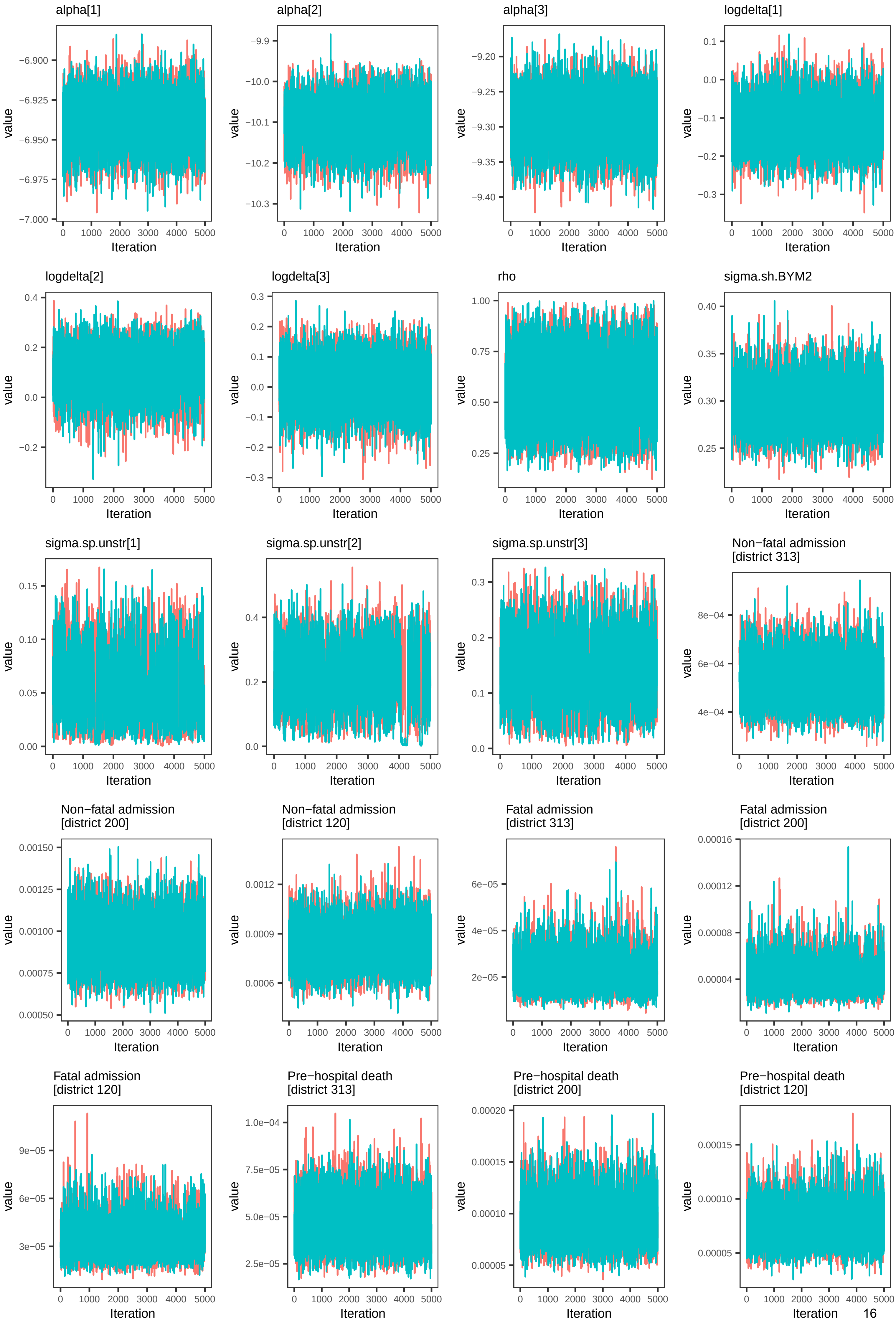

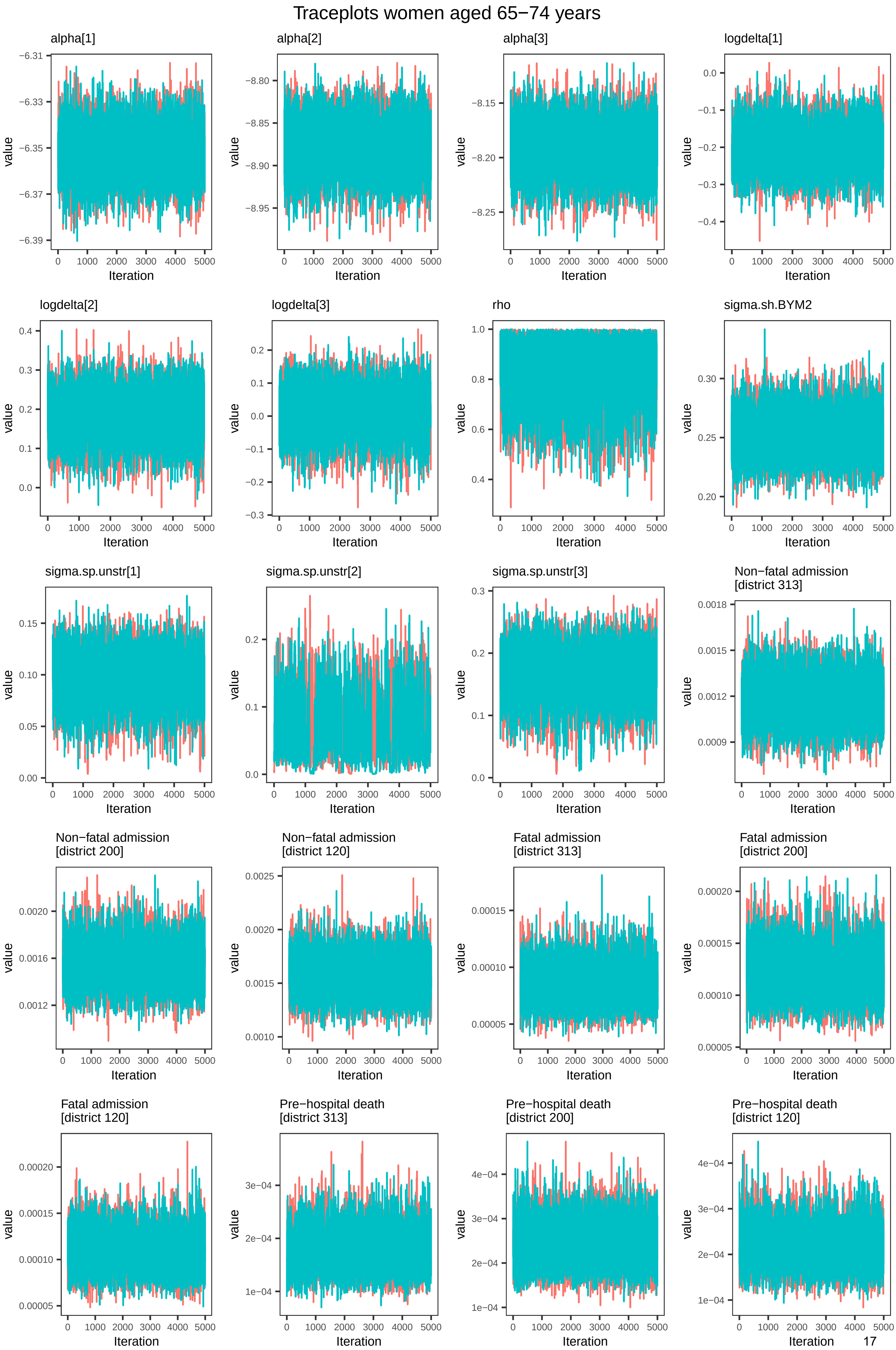

Traceplots women aged 65−74 years
alpha[1]
alpha[2]
alpha[3]
logdelta[1]
logdelta[2]
logdelta[3]
rho
sigma.sh.BYM2
sigma.sp.unstr[1]
sigma.sp.unstr[2]
sigma.sp.unstr[3]
Non−fatal admission [district 313]
Non−fatal admission [district 200]
Non−fatal admission [district 120]
Fatal admission [district 313]
Fatal admission [district 200]
Fatal admission [district 120]
Pre−hospital death [district 313]
Pre−hospital death [district 200]
Pre−hospital death [district 120]
value
Iteration

Traceplots women aged 75−84 years

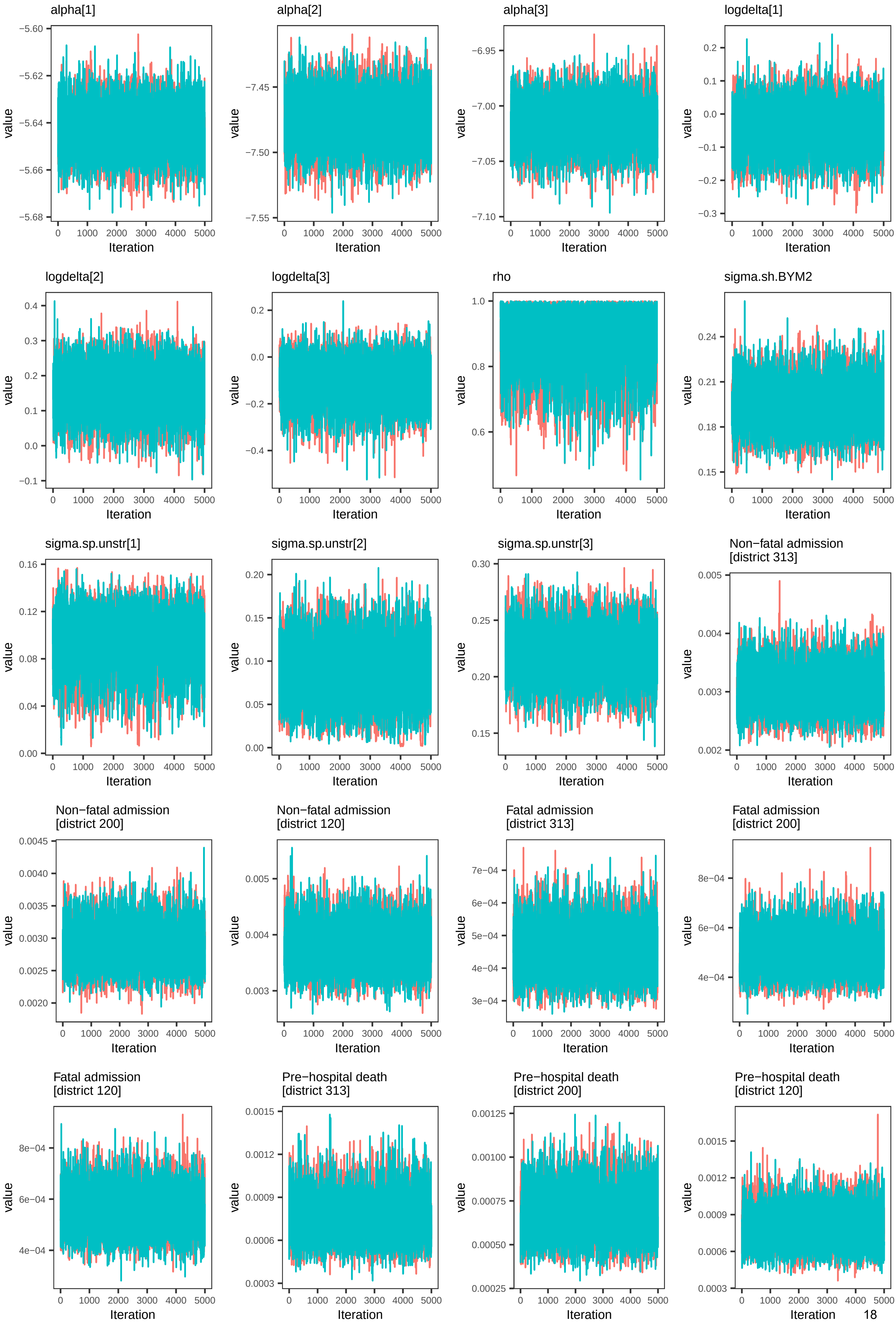

Traceplots women aged 85+ years

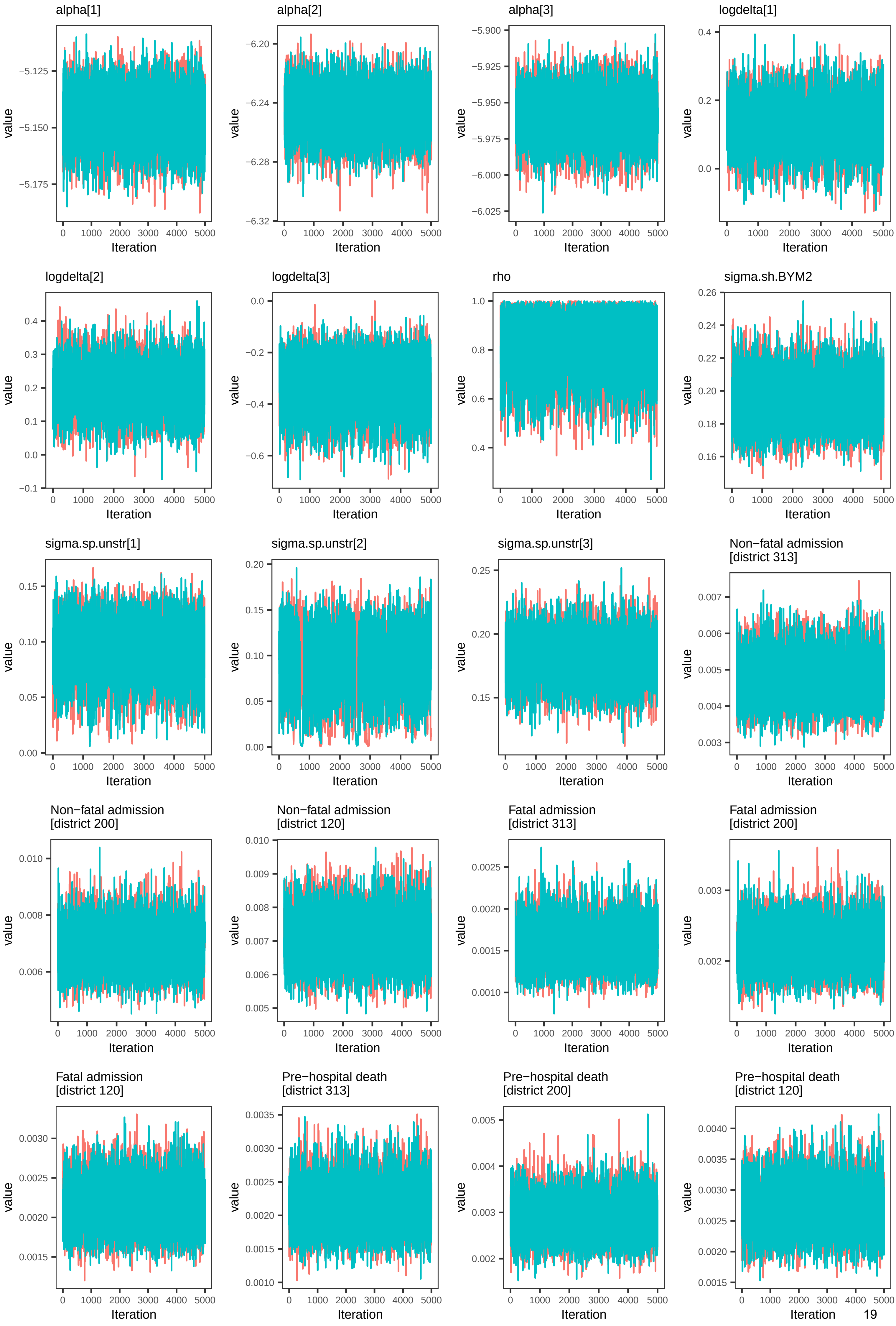

Traceplots men aged 45−54 years

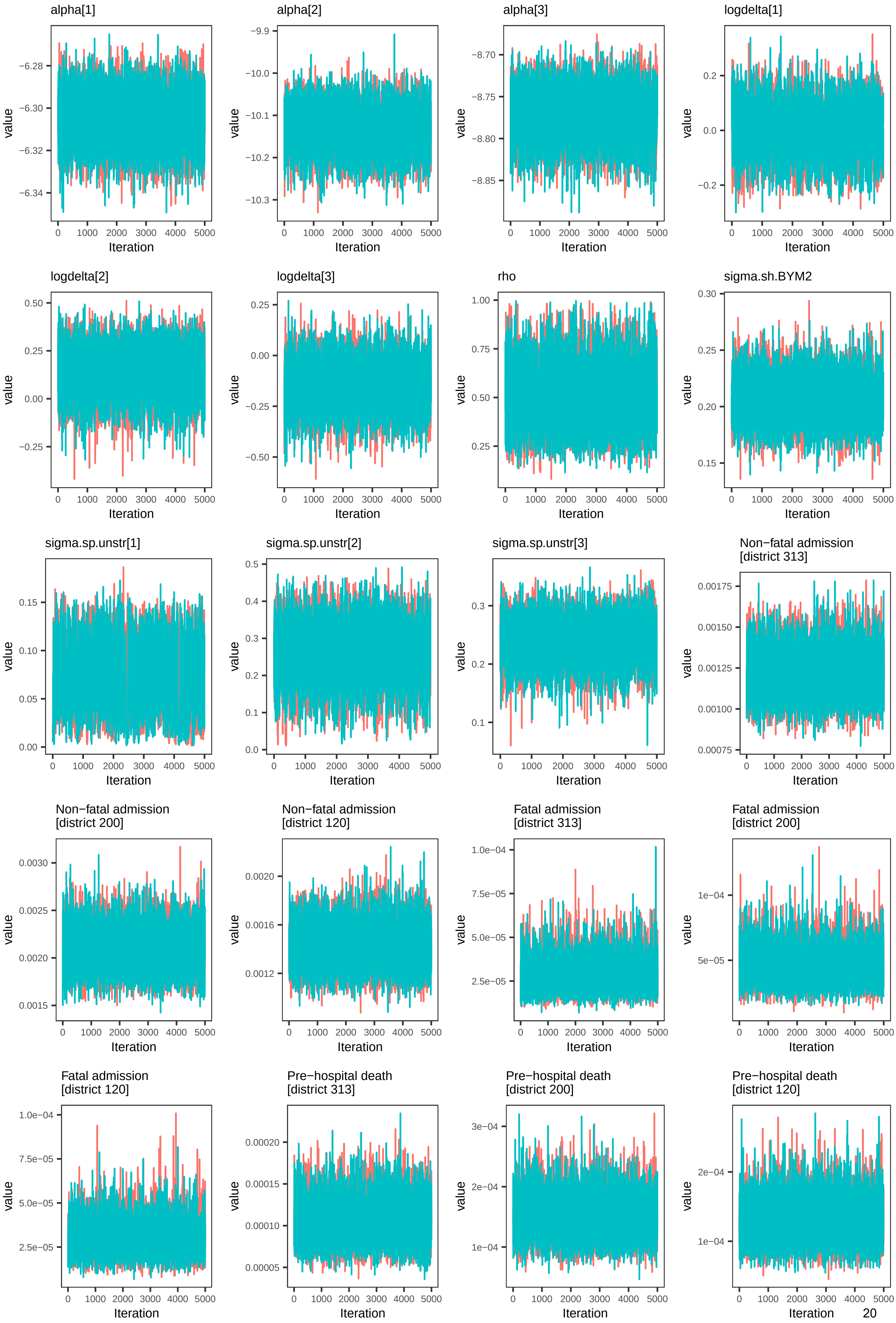

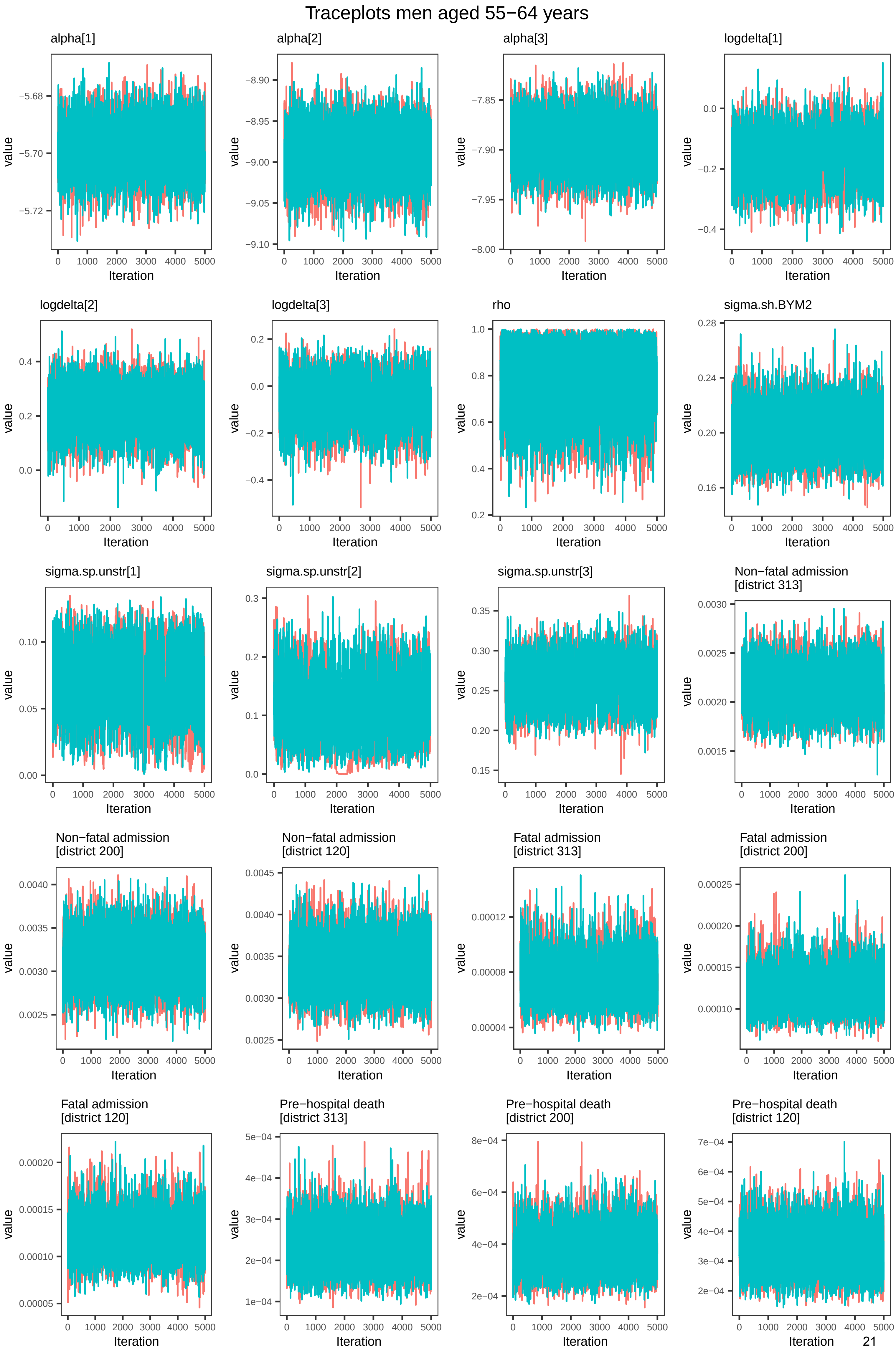
Traceplots men aged 55−64 years
alpha[1]
alpha[2]
alpha[3]
logdelta[1]
logdelta[2]
logdelta[3]
rho
sigma.sh.BYM2
sigma.sp.unstr[1]
sigma.sp.unstr[2]
sigma.sp.unstr[3]
Non−fatal admission [district 313]
Non−fatal admission [district 200]
Non−fatal admission [district 120]
Fatal admission [district 313]
Fatal admission [district 200]
Fatal admission [district 120]
Pre−hospital death [district 313]
Pre−hospital death [district 200]
Pre−hospital death [district 120]
value
Iteration

Traceplots men aged 65−74 years

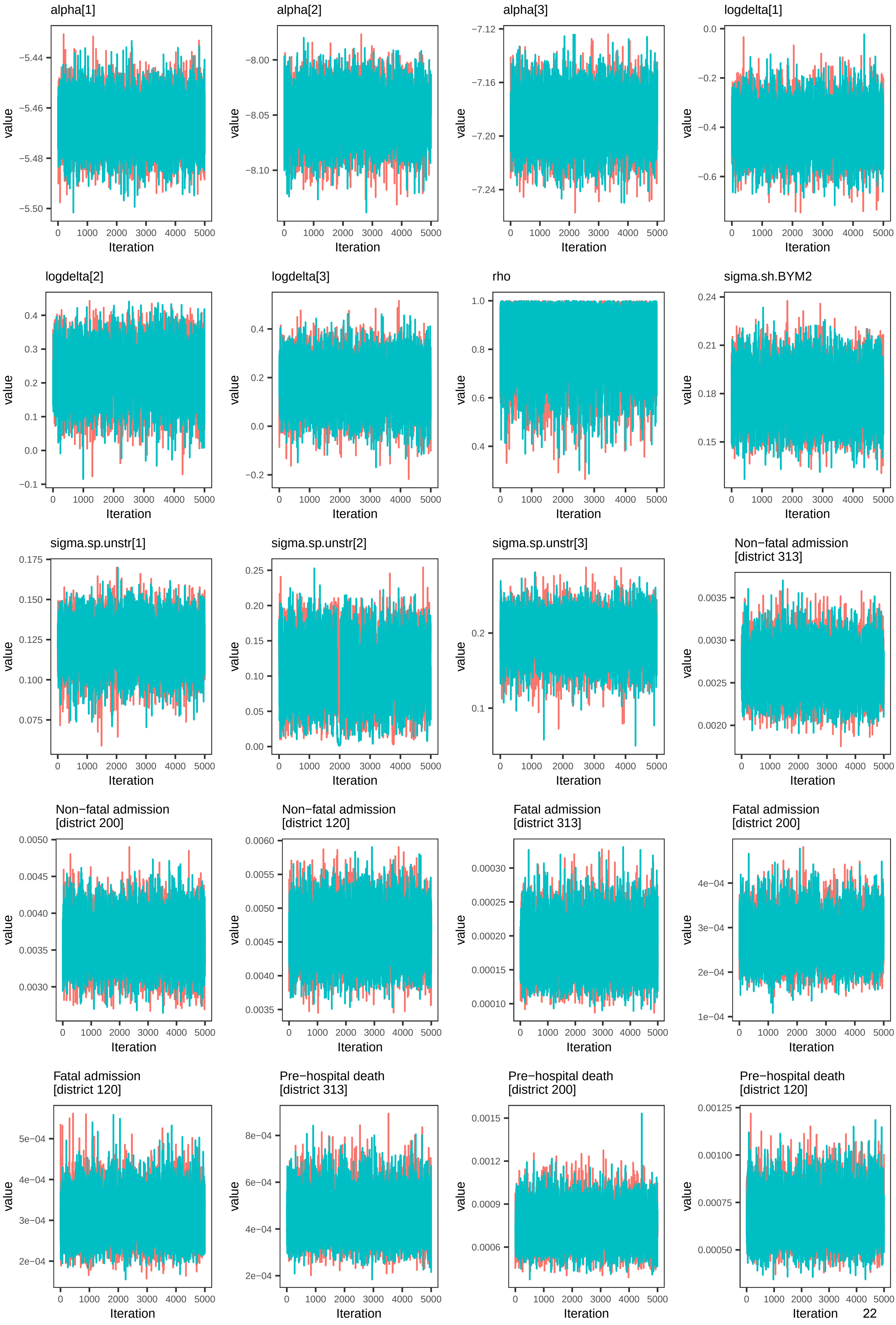

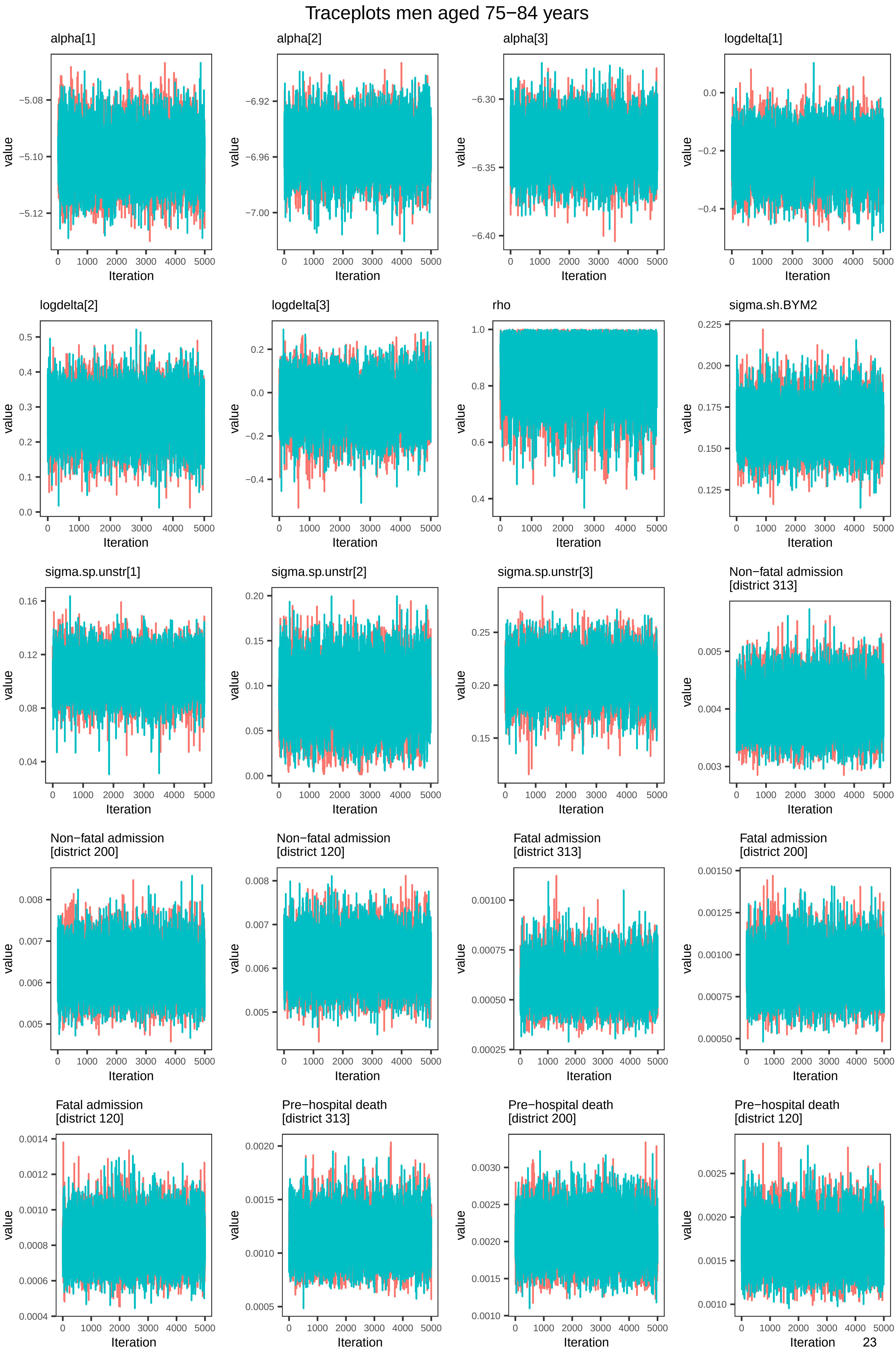

Traceplots men aged 75−84 years
alpha[1]
alpha[2]
alpha[3]
logdelta[1]
logdelta[2]
logdelta[3]
rho
sigma.sh.BYM2
sigma.sp.unstr[1]
sigma.sp.unstr[2]
sigma.sp.unstr[3]
Non−fatal admission [district 313]
Non−fatal admission [district 200]
Non−fatal admission [district 120]
Fatal admission [district 313]
Fatal admission [district 200]
Fatal admission [district 120]
Pre−hospital death [district 313]
Pre−hospital death [district 200]
Pre−hospital death [district 120]
value
Iteration

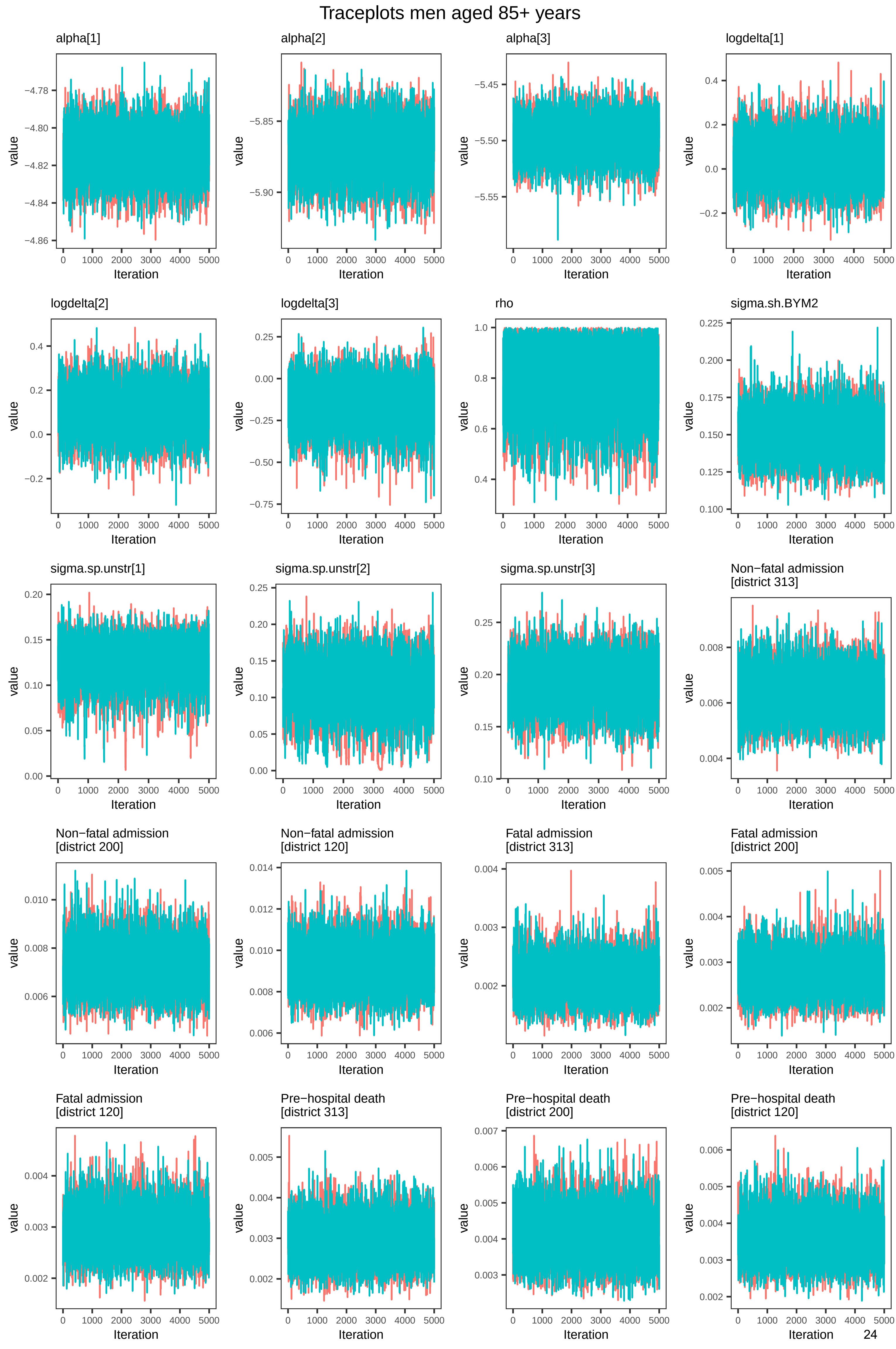
Traceplots men aged 85+ years
alpha[1]
alpha[2]
alpha[3]
logdelta[1]
logdelta[2]
logdelta[3]
rho
sigma.sh.BYM2
sigma.sp.unstr[1]
sigma.sp.unstr[2]
sigma.sp.unstr[3]
Non−fatal admission [district 313]
Non−fatal admission [district 200]
Non−fatal admission [district 120]
Fatal admission [district 313]
Fatal admission [district 200]
Fatal admission [district 120]
Pre−hospital death [district 313]
Pre−hospital death [district 200]
Pre−hospital death [district 120]
value
Iteration

References

1. Besag J, York J, Mollie A. Bayesian image restoration, with two applications in spatial statistics (with discussion). *Ann I Stat Math* 1991; **43**(1): 1-59.
2. Riebler A, Sorbye SH, Simpson D, Rue H. An intuitive Bayesian spatial model for disease mapping that accounts for scaling. *Stat Methods Med Res* 2016; **25**(4): 1145-65.
3. Freni-Sterrantino A, Ventrucci M, Rue H. A note on intrinsic conditional autoregressive models for disconnected graphs. *Spat Spatiotemporal Epidemiol* 2018; **26**: 25-34.
4. Rashid T, Bennett JE, Paciorek CJ, et al. Life expectancy and risk of death in 6791 communities in England from 2002 to 2019: high-resolution spatiotemporal analysis of civil registration data. *The Lancet Public Health* 2021; **6**(11): e805-e16.
5. Bennett JE, Li G, Foreman K, et al. The future of life expectancy and life expectancy inequalities in England and Wales: Bayesian spatiotemporal forecasting. *Lancet* 2015; **386**(9989): 163-70.
6. Foreman KJ, Li G, Best N, Ezzati M. Small area forecasts of cause-specific mortality: application of a Bayesian hierarchical model to US vital registration data. *Journal of the Royal Statistical Society: Series C (Applied Statistics)* 2017; **66**(1): 121-39.
7. Knorr-Held L, Best N. A shared component model for detecting joint and selective clustering of two diseases. *Journal of the Royal Statistical Society A* 2001; **164**: 73-85.
8. Best N, Hansell AL. Geographic variations in risk: adjusting for unmeasured confounders through joint modeling of multiple diseases. *Epidemiology (Cambridge, Mass)* 2009; **20**(3): 400.
9. Mahaki B, Mehrabi Y, Kavousi A, Schmid VJ. Joint spatio-temporal shared component model with an application in Iran Cancer Data. *Asian Pacific journal of cancer prevention: APJCP* 2018; **19**(6): 1553.
10. Downing A, Forman D, Gilthorpe MS, Edwards KL, Manda SO. Joint disease mapping using six cancers in the Yorkshire region of England. *International journal of health geographics* 2008; **7**(1): 1-14.
11. Oleson JJ, Smith BJ, Kim H. Joint spatio-temporal modeling of low incidence cancers sharing common risk factors. *J Data Sci* 2008; **6**: 105-23.
12. de Valpine P, Turek D, Paciorek CJ, Anderson-Bergman C, Lang DT, Bodik R. Programming With Models: Writing Statistical Algorithms for General Model Structures With NIMBLE. *Journal of Computational and Graphical Statistics* 2017; **26**(2): 403-13.
13. NIMBLE Development Team. NIMBLE: MCMC, Particle Filtering, and Programmable Hierarchical Modeling. Version 0.12.1. Available at https://doi.org/10.5281/zenodo.5562925, 2021.
14. Brooks SP, Gelman A. General methods for monitoring convergence of iterative simulations. *Journal of Computational and Graphical Statistics* 1998; **7**(4): 434-55.
15. Lunn DJ, Thomas A, Best N, Spiegelhalter D. WinBUGS - A Bayesian modelling framework: Concepts, structure, and extensibility. *Stat Comput* 2000; **10**(4): 325-37.
